# A systematic review of deep learning methods for community detection in social networks

**DOI:** 10.3389/frai.2025.1572645

**Published:** 2025-08-22

**Authors:** Mohamed El-Moussaoui, Mohamed Hanine, Ali Kartit, Monica Garcia Villar, Helena Garay, Isabel de la Torre Díez

**Affiliations:** ^1^LTI Laboratory, Department of Telecommunications, Networks, and Informatics, ENSA, Chouaib Doukkali University, El Jadida, Morocco; ^2^Universidad Europea del Atlantico, Santander, Spain; ^3^Universidad Internacional Iberoamericana, Campeche, Mexico; ^4^Universidad Internacional Iberoamericana, Arecibo, Puerto Rico; ^5^Universidad International do Cuanza, Kuito, Angola; ^6^Universidad de La Romana, La Romana, Dominican Republic; ^7^Department of Signal Theory and Communications and Telematic Engineering, University of Valladolid, Valladolid, Spain

**Keywords:** community detection, graph clustering, deep learning techniques, systematic literature review (SLR), PICO framework

## Abstract

**Introduction:**

The rapid expansion of generated data through social networks has introduced significant challenges, which underscores the need for advanced methods to analyze and interpret these complex systems. Deep learning has emerged as an effective approach, offering robust capabilities to process large datasets, and uncover intricate relationships and patterns.

**Methods:**

In this systematic literature review, we explore research conducted over the past decade, focusing on the use of deep learning techniques for community detection in social networks. A total of 19 studies were carefully selected from reputable databases, including the ACM Library, Springer Link, Scopus, Science Direct, and IEEE Xplore. This review investigates the employed methodologies, evaluates their effectiveness, and discusses the challenges identified in these works.

**Results:**

Our review shows that models like graph neural networks (GNNs), autoencoders, and convolutional neural networks (CNNs) are some of the most commonly used approaches for community detection. It also examines the variety of social networks, datasets, evaluation metrics, and employed frameworks in these studies.

**Discussion:**

However, the analysis highlights several challenges, such as scalability, understanding how the models work (interpretability), and the need for solutions that can adapt to different types of networks. These issues stand out as important areas that need further attention and deeper research. This review provides meaningful insights for researchers working in social network analysis. It offers a detailed summary of recent developments, showcases the most impactful deep learning methods, and identifies key challenges that remain to be explored.

## 1 Introduction

In recent years, the exponential utilization of social networks has generated an immense volume of data, making the field of social network analysis (SNA) an important resource for understanding the structures and the dynamic of these social networks. As part of the foundation of SNA, community detection focuses on identifying groups of nodes within a network, where nodes representing users or entities more densely connected among themselves than with the rest of the network components. Community identification holds critical importance across diverse domains, including sociology, transportation, marketing, finance and security and so on, as it enables the identification of interaction patterns, influence dynamics, and shared attributes among network components. However, as social networks become increasingly complex, traditional community detection methods face significant limitations, particularly in addressing challenges related to scalability, precision, and adaptability (Fortunato, 2010).

The emergence of deep learning has revolutionized various domains of data analysis, including the field of community detection within social networks. Through the utilization of advanced models such as graph neural networks (GNNs), convolutional neural networks (CNNs), and autoencoders, deep learning techniques have demonstrated remarkable capabilities in capturing complex and non-linear relationships within large-scale networks (Wang et al., 2015). These models excel in automatically learning meaningful representations of the network components, uncovering hidden structures and patterns that are often out of reach of classical algorithms. Traditional community detection methods such as modularity optimization, label propagation, or spectral clustering, typically rely on predefined topological heuristics and are often limited in their ability to handle overlapping communities, dynamic structures, and high-dimensional node attributes. In contrast, deep learning approaches provide a data-driven framework that enables the extraction of richer, multi-scale representations, offering greater flexibility and adaptability to modern network analysis tasks.

Furthermore, deep learning techniques demonstrate adaptability to dynamic and evolving networks, enabling more precise identification of community structures (Kipf and Welling, 2016). The application of deep learning to community detection offers several key advantages. First, deep learning models can process high-dimensional, heterogeneous data, making them well-suited for social networks where interactions between nodes can be based on multiple factors (Veličković et al., 2017). Second, these models are capable of learning from both network structure and node features, providing a more comprehensive understanding of community dynamics. Finally, the flexibility of deep learning enables the integration of unsupervised, semi-supervised (Berahmand et al., 2023), and supervised learning techniques, offering robust solutions in different network environments (Li et al., 2017).

Despite the promising potential of deep learning in community detection, several challenges and open questions still persist. One significant challenge is the interpretability of deep learning models. Although these models often achieve good performances, their complexity makes it difficult to understand the reasoning behind specific community assignments, limiting their transparency. The second major challenge is the scalability of deep learning approaches for extremely large networks. Training and inference in these models often require important computational resources, which creates a barrier to their application in massive networks (Li et al., 2017). Furthermore, the dynamic nature of social networks creates an additional layer of complexity. Models must be capable of continuously adapting to new data and structural changes, which remains an active field of research (Wu et al., 2020; Pan et al., 2024; Li X. et al., 2023).

This systematic literature review (SLR) synthesizes the state-of-the-art research on the application of deep learning methods for community detection in social networks. By analyzing key trends, techniques, and challenges, this review aims to provide a comprehensive understanding of how deep learning is shaping this field and identify areas for future research. We followed a rigorous and repeatable methodology to review 19 papers published between 2014 and 2024, extracted from ACM Library, Springer Link, Scopus, Science Direct and IEEE. Our focus encompasses a wide range of deep learning techniques, from graph-based models to deep unsupervised learning approaches, as well as the open challenges in this evolving domain.

The remainder of this paper is structured as follows: Section 2 presents related works in the area of community detection and deep learning. Section 3 details the research methodology used in this review. Section 4 presents the results of our analysis. Section 5 discusses the findings in depth, and Section 6 addresses the limitations of this study and potential directions for future work. Finally, Section 7 concludes the paper by summarizing the key contributions and insights.

## 2 Related work

Community detection has been a long-standing area of research, with early approaches relying on graph-theoretic techniques such as modularity maximization, hierarchical clustering, and spectral clustering (Fortunato, 2010; Berahmand et al., 2018, 2025). While foundational, these methods often struggle with scalability and adaptability to dynamic, large-scale networks. Recent advancements such as AGNMF-AN (Berahmand et al., 2022) have extended traditional spectral methods to handle attributed networks by integrating structural and attribute information more effectively.

With the emergence of deep learning, more flexible and scalable models have been proposed. Techniques such as Graph Neural Networks (GNNs), Convolutional Neural Networks (CNNs), and autoencoders have enabled automatic extraction of meaningful node representations, supporting more accurate and robust community detection in complex social networks (Wang et al., 2015; Lin and Kang, 2021). GNNs in particular have gained popularity due to their ability to learn from both local and global graph structures. Notable variants include Graph Convolutional Networks (GCNs) (Kipf and Welling, 2016; Hong et al., 2020) and Graph Attention Networks (GATs) (Veličković et al., 2017), which introduce attention mechanisms to weigh node importance adaptively.

Autoencoders and variational autoencoders (VAEs) are also widely applied to reduce dimensionality while preserving network structure, facilitating clear community separations (Li et al., 2017; Shi et al., 2019). The recent DSSC framework (Berahmand et al., 2023) enhances semi-supervised detection by incorporating PMI-based side information filtering, while SDAC-DA (Berahmand et al., 2024) jointly optimizes structure and attribute consistency in attributed graphs.

For dynamic or evolving networks, Deep Reinforcement Learning (DRL) offers a promising paradigm. For instance, the method proposed in Wu et al. (2020) uses DRL to refine communities in real time by optimizing metrics like modularity in changing environments.

In addition to standalone DL methods, hybrid approaches have emerged that combine traditional clustering techniques (e.g., spectral clustering, K-means) with deep models such as Deep Belief Networks (DBNs) or autoencoders (Dhilber and Bhavani, 2020; Li H. et al., 2023). These approaches aim to balance interpretability and performance by leveraging the strengths of both paradigms.

Several recent systematic literature reviews (SLRs) have surveyed related topics—such as DL in social networks (Catal et al., 2022; Li C. et al., 2023), hybrid community detection (Şencan et al., 2022), and comprehensive analyses of community detection techniques (Su et al., 2022) as summarized in Table 1. However, few focus specifically on deep learning approaches for community detection. Our review addresses this gap by (i) restricting analysis to DL-based techniques, (ii) prioritizing reproducibility and benchmarking rigor, and (iii) covering underexplored trends such as dynamic graphs and self-supervised learning.

**Table 1:** Comparison of related systematic reviews.

**SLR**	**Year**	**Type**	**# Papers**	**Timespan**	**Database sources**
Catal et al. (2022)	2024	Journal	40	2015–2020	ACM, IEEE Xplore, ScienceDirect, Springer, Wiley
Li C. et al. (2023)	2023	Journal	53	2018–2023	Scopus, Web of Science, IEEE Xplore, EBSCOHost, SpringerLink, ScienceDirect
Şencan et al. (2022)	2022	Journal	20/83	2016–2022	IEEE Xplore, ScienceDirect
Rostami et al. (2023)	2023	Journal	45	2018–2022	IEEE Xplore
Su et al. (2022)	2021	Journal	168	2005–2021	DBLP, ACM

## 3 Research methodology

### 3.1 Review questions

To conduct a systematic literature review (SLR), it is essential to develop precise and clear research questions that would guide the scope, and focus of this SLR review. The process begins with the formulation of well-defined review questions, which establishes the foundation for identifying, selecting, and synthesizing relevant studies. The established SLR protocols were followed in this review to ensure methodological rigor, transparency, and replicability. The process was structured to maintain a high standard of comprehensiveness and accuracy, starting with the development of inclusion and exclusion criteria for systematic selection of studies.

In fact, the PICO framework (Amir-Behghadami and Janati, 2020), a widely recognized approach in systematic reviews, was employed to construct a robust and targeted search strategy. Through this framework, we can develop research questions and queries that cover studies related to the intersection of deep learning techniques and community detection methods. The objectives of this systematic review were achieved by capturing a diverse and relevant set of studies.

In addition, a rigorous screening procedure was employed to select studies that align with our research objectives, which enables us to collect a set of studies that significantly enhance our understanding of how deep learning methods are used and evaluated within the field of social networks.

The following key-items describe the addressed questions:

- RQ1: What are the most commonly used deep learning techniques in community detection? This question aims to identify the deep learning methods that are most frequently applied to the problem of community detection. By analyzing the techniques and architectures that dominate the field, this question will offer insights into the existing approaches and their effectiveness in addressing the challenges of community detection in social networks.
- RQ2: How are deep learning models for community detection structured and documented in the literature? This question investigates the scope to which deep learning models for community detection are explicitly described in the literature. A detailed analysis of how well these models are documented can help determine their reproducibility and ease of implementation for future researchers.
- RQ3: What types of graph or network structures are primarily addressed in deep learning-based community detection? This question explores the types of network datasets that are most commonly used in community detection studies. Seeking to understand how well deep learning approaches generalize across different graph structures.
- RQ4: What are the key performance metrics used to evaluate deep learning models for community detection? This question aims to identify the most frequently used evaluation metrics in studies that apply deep learning to community detection. It will help assess how the effectiveness of these models is measured and compared across different research works.
- RQ5: What are the most prominent machine learning and hybrid approaches used alongside deep learning for community detection? This question aims to explore how deep learning is combined with other machine learning techniques, or hybrid approaches, in community detection. It will clarify on the complementary methods that enhance the performance of the used deep learning models.
- RQ6: What datasets are commonly used to train and evaluate deep learning models for community detection? This question focuses on identifying the publicly available datasets most commonly used in the training and evaluation of deep learning models for community detection.
- RQ7: What tools and frameworks are utilized for implementing deep learning models in community detection? This question seeks to reveal the software tools, libraries, and frameworks that are commonly used to implement and experiment. Understanding this technological landscape will help researchers in selecting the appropriate tools.
- RQ8: What are the common challenges and limitations faced in applying deep learning to community detection? This question aims to identify the recurrent challenges, both technical and practical, that prevent the effective application of deep learning to community detection. It will highlight open research problems and areas that require further investigation or innovation.
- RQ9: What are the emerging trends in the application of deep learning to community detection? This question investigates the latest trends in research on deep learning for community detection, focusing on innovative techniques, novel applications, and evolving methodologies. It will provide a perspective view of the future developments in the field.
- RQ10: How do the domains or types of networks influence the effectiveness of deep learning in community detection? This question explores the specific domains where deep learning has shown the most success or encountered the most difficulties. Which will help identify domain-specific challenges, and the need for customized solutions based on network characteristics.

We provide the key review questions that support this SLR and guide the investigation process. The aim behind the above questions is to explore the state-of-the-art in using deep learning methods for community detection, evaluate their effectiveness, and identify gaps in the current literature. The proposed review is structured around these research questions to ensure that our analysis remains focused on the major topics of analysis while providing a comprehensive overview of the contributions, challenges, and opportunities in this field.

The proposed research questions were classified to demonstrate their effectiveness. Using this classification, the questions are properly structured to guide the review process and provide clarity on technical, methodological, and trend-related aspects of the research. The SLR addresses both theoretical and practical aspects of community detection using deep learning techniques following a structured approach to formulating review questions. Table 2 summarizes the results of the classification and purpose of the questions.

**Table 2:** Categorization and purpose of questions defined for the SLR review.

**Category**	**RQs**	**Purpose**
Technical approach/algorithm	RQ1, RQ5	Identify the deep learning methods and hybrid machine learning approaches used in community detection. This provides a foundation for understanding the current state-of-the-art and technical landscape in the field.
Model structure and reproducibility	RQ2	Evaluating how well models are described and their reproducibility is crucial for assessing the usability and reliability of the proposed techniques. This ensures that methods are sufficiently documented for reuse by other researchers.
Graph and network types	RQ3, RQ10	Explore the types of graphs and networks where the models are applied, including the domain-specific challenges. This ensures the review covers the diversity of applications and addresses specialized needs.
Evaluation metrics	RQ4	Understanding the performance metrics, such as accuracy, efficiency, precision, and recall, is essential for comparing studies and identifying key priorities in evaluating the models.
Datasets	RQ6	Datasets are a critical part of deep learning research. These questions focus on identifying the datasets most frequently used in the field, helping to evaluate the generalization potential of models and ensuring a fair comparison across studies.
Tools and frameworks	RQ7	Highlighting the tools and frameworks employed in the research aids practitioners and researchers in selecting the most effective platforms for implementation. This also provides insight into emerging technological trends.
Challenges and limitations	RQ8	Identifying open problems, bottlenecks, and limitations in the field helps uncover research gaps. These questions guide further exploration of unresolved issues and point to areas where innovation is needed.
Emerging trends	RQ9	These questions seek to understand the evolving trends and developments in deep learning for community detection, ensuring the review remains forward-looking and relevant to ongoing advances in the field.

### 3.2 Inclusion and exclusion criteria

The inclusion and exclusion criteria are clarified in this section, ensuring that the SLR review globally concentrates on the most relevant and high-quality studies. By excluding studies that are not fully or partially aligned with the research objectives. The establishment of those criteria (Table 3) guides the selection of papers for analysis, ensuring both comprehensiveness and relevance in addressing the definitional questions.

**Table 3:** Inclusion and exclusion criteria.

**Criterion**	**Description**
**Inclusion criteria**	
Publication date	Studies published after 2014 are included to ensure the review captures recent advancements in deep learning for community detection.
Peer-reviewed journal articles	Only peer-reviewed journal articles are included to ensure quality and reliability of the findings.
Language	Only English-language studies are included, as English is the predominant scholarly language.
Focus on deep learning for community detection	Papers must focus on community detection, graph clustering, or community identification using deep learning (DL), machine learning (ML), or hybrid methods.
Empirical evaluations	Papers must include thorough evaluation of methods, using established datasets or experimental setups, to assess model performance.
Relevance to social networks	Only papers with practical insights or real-world applications related to community detection in social networks are included.
Reproducibility requirements	Studies must provide sufficient methodological detail for experimental replication (e.g., hyperparameters, training protocols).
**Exclusion criteria**	
Pre-2014 studies	Papers published before 2014 will be excluded, as they may not reflect current deep learning developments.
Non-deep learning approaches	Studies focused solely on traditional algorithms (e.g., modularity-based methods, spectral clustering) without DL and ML application are excluded.
Conference papers and non-academic publications	Conference papers, technical reports, and non-peer-reviewed articles are excluded to maintain a high-quality dataset.
Non-English papers	Papers published in languages other than English are excluded to avoid language barriers in interpretation.
Irrelevant applications	Papers not focused on community detection (e.g., link prediction, node classification) will be excluded unless community detection is a primary task.
Incomplete studies or early access papers	Incomplete studies or early access papers that have not undergone full peer-review will be excluded.
Non-reproducible studies	Papers lacking sufficient methodological detail or implementation information for replication.

#### 3.2.1 Database selection

In this section, we gathered a diverse collection of high-quality research papers using reputable academic databases. The databases that were chosen are SCOPUS, SpringerLink, ScienceDirect, ACM Digital Library, and IEEE Xplore, all of which are well-known for their extensive coverage of scientific literature. By using this approach, we can gain valuable insights into the current trends and developments at the intersection of deep learning techniques and community detection methods.

#### 3.2.2 Search strategy

The review framework we use to structure our search query is described in this section, to ensure that the studies retrieved are both precise and relevant.

The PICOS framework, an extension of the traditional PICO model, was adopted to ensure a structured and systematic approach to the review process. Standing for Population, Intervention, Comparison, Outcomes, and Study design, each component of the PICOS framework was specifically adapted to align with the objectives of this study. In this context, the Population represents studies applying community detection in social networks. While the Intervention focuses on deep learning-based community detection techniques. Where the Comparison considers alternative algorithms and methods within the literature. Then, the Outcomes look for advancements in effectiveness or performance metrics, and Study design emphasizes peer-reviewed journal articles published in English.

Using PICOS framework provides several advantages for this SLR, especially in filtering literature to align with the study's objectives. By clearly defining each component of PICOS, we ensured a focused search query that minimizes irrelevant results and emphasizes high-quality research contributions. Furthermore, the PICOS approach strengthens the replicability of the review by applying a structured and consistent method to the formulation, inclusion, and exclusion criteria of the query, aligning well with our objective to provide a comprehensive synthesis of community detection methods using deep learning across social networks.

To ensure comprehensive and systematic identification of relevant studies on community detection using deep learning or machine learning techniques, a structured search query was formulated and executed in each of the selected databases.

The search query was designed to retrieve papers that focus on community detection, graph clustering, or community identification, while employing machine learning or deep learning techniques. Specifically, the following keywords and search operators were used:

- Keywords for the problem domain: we included the terms: “Community detection,” “Graph clustering,” and “Community identification” to capture all relevant studies that investigate community structures within networks. These keywords represent common terminology in both graph theory and social network analysis.
- Keywords for the employed methods: to focus on papers using machine learning techniques, the search included terms such as “Deep learning” and “Machine learning.” Which were used to ensure that the studies in the final dataset apply advanced learning methods to solve community detection problems.

In constructing our search queries, we employed the NEAR logical operator to enhance the precision of the results. The NEAR operator ensures that the specified above keywords appear in close proximity to each other. This proximity-based matching is crucial for capturing papers where these concepts are tightly related and discussed within the same context. Unlike broader operators like AND, which may retrieve results where the terms appear in unrelated sections of a paper, NEAR improves the relevance of the search by focusing on articles where the concepts of community detection and deep learning are directly linked.

This approach effectively retrieves studies that are more likely to align with the focus of our systematic review, leading to more accurate and contextually meaningful results.

- Keywords in title, abstract, or keywords (TITLE-ABS-KEY): a constructed query to search for articles where the specified terms appeared in either the title, abstract, or keywords fields.
- Publication year (PUBYEAR > 2014): a restricted the search on papers published after 2014, ensuring that only last decade contributions were included.
- Document type [LIMIT-TO (DOCTYPE, “ar”)]: the search was limited to “ar” (article), meaning only peer-reviewed journal articles were considered.
- Language [LIMIT-TO (LANGUAGE, “English”)]: to avoid potential language barriers in interpreting the results, the search was restricted to studies written and published in English language.
- Source type [LIMIT-TO (SRCTYPE, “j”)]: the search was further restricted to journals, excluding conference proceedings, books, and other sources to focus on in-depth studies typically found in journal articles.
- Publication STAGE [LIMIT-TO (PUBSTAGE, “final”)]: the search was limited to papers that have reached the final publication stage, ensuring that only completed and officially published studies were included.

This search strategy allowed us to obtain a curated set of papers that are relevant, peer-reviewed, and methodologically robust, laying a strong foundation for our literature review on community detection using deep learning approaches.

#### 3.2.3 Inclusion/exclusion steps

**Figure 1:**
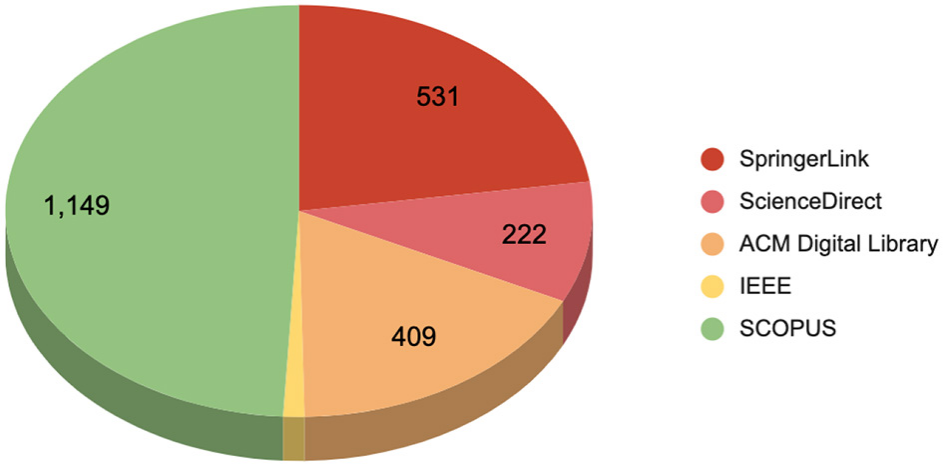
The total extracted articles per database.

**Figure 2:**
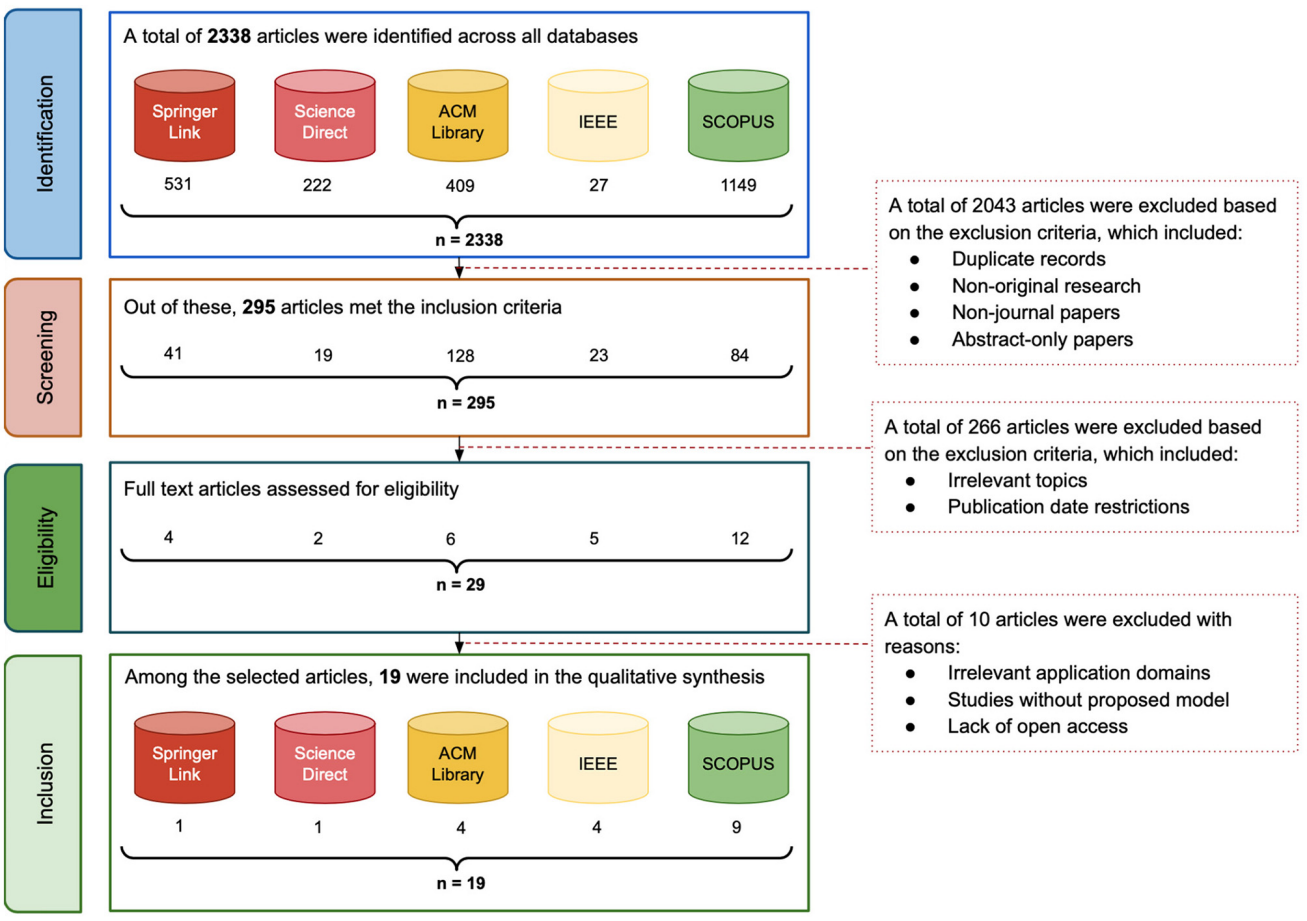
Flow diagram of study inclusion and exclusion criteria.

Once the formulated search queries and relevant databases are identified, we executed the query on SCOPUS, SpringerLink, ScienceDirect, ACM Digital Library, and IEEE Xplore, providing an initial dataset of 2,338 publications. The subsequent screening process involved the removal of duplicate recods, exclusion of non-English papers, filtering of articles published outside the 2014–2024 range, and elimination of studies not conforming to our focus on peer-reviewed journal articles. We illustrate in Figure 1 the total number of retrieved papers per database, where the Figure 2 illustrates a flowchart of the inclusion and exclusion criteria applied during this process.

### 3.3 Quality assessment

The quality assessment of the selected studies is an important element in this SLR. It was designed to ensure that the conclusions are drawn from reliable and well-documented research. This step emphasizes supporting methodological standards and examining the latest advancements in deep learning techniques for community detection. A rigorous evaluation framework is utilized to carefully analyze factors such as (i) the clarity of research objectives, (ii) the relevance of methodologies, and (iii) the depth of analysis. Each study is examined in this analysis, which offers a transparent and thorough evaluation of their validity and contribution to the field. This strategy not only enhances the interpretation of findings but also identifies critical gaps in the current research framework.

All selected studies included in this review were evaluated in detail to highlight their strengths and identify any limitations. This process helps evaluating the contributions within the context of the predefined research objectives. A rigorous and standardized quality assessment approach was employed to enhance the credibility of the findings, offering a clear understanding of the validity of the conclusions.

The assessment criteria focused on the following questions to ascertain the quality of the primary studies:

- Does the paper utilize deep learning techniques specifically for community detection? This criterion ensures that the focus remains on studies leveraging deep learning as opposed to solely traditional or hybrid approaches.
- Is the methodology clearly defined, including the problem statement, the selection of deep learning algorithms, and the approach's architecture? Clarity in the presentation of the problem, solution approach, and deep learning technique is essential for understanding the applicability and reproducibility of the study.
- Is there empirical evidence for the proposed approach? The presence of experimental results, case studies, or evaluation outcomes is critical for assessing the practical performance and effectiveness of the deep learning methods used.

After applying both the inclusion/exclusion criteria and quality assessment measures, the SLR included a refined set of high-quality studies. This deliberate selection ensured that only studies providing comprehensive insights, empirical evaluations, and detailed applications of deep learning techniques in community detection were retained. The emphasis on high-quality, full-implementation studies reinforces the credibility of this SLR and allows for a reliable synthesis of key findings and an exploration of recurring themes and challenges in the field.

This assessment framework helps maintain the integrity of the review, enabling a focused analysis that informs future research and practical applications in community detection using deep learning.

### 3.4 Paper analysis

After applying the defined inclusion/exclusion criteria and conducting a quality assessment, we performed an in-depth analysis of the remaining 19 papers to gather relevant information for answering the review questions related to community detection in social networks using deep learning techniques. This extracted information was systematically organized into a spreadsheet, with the data classified into various columns, as illustrated in Table 2.

As a critical analysis of the 19 selected studies revealed significant methodological variations:

- Only 42% provided complete implementation details, with just Klepper et al. (2024) and Wu et al. (2020) offering containerized solutions;
- Testing scales varied dramatically, with 73% evaluating on small graphs (Less than 100K nodes) while only Levie et al. (2019) addressed billion-edge networks;
- Benchmarking practices showed inconsistencies, as 79% used are under three baselines and none included recent graph transformers;
- Evaluation rigor was compromised by metric heterogeneity where 89% of the selected papers mixing modularity with clustering metrics without normalization and limited temporal testing in five studies using incompatible time-window approaches.

### 3.5 Final papers dataset

**Figure 3:**
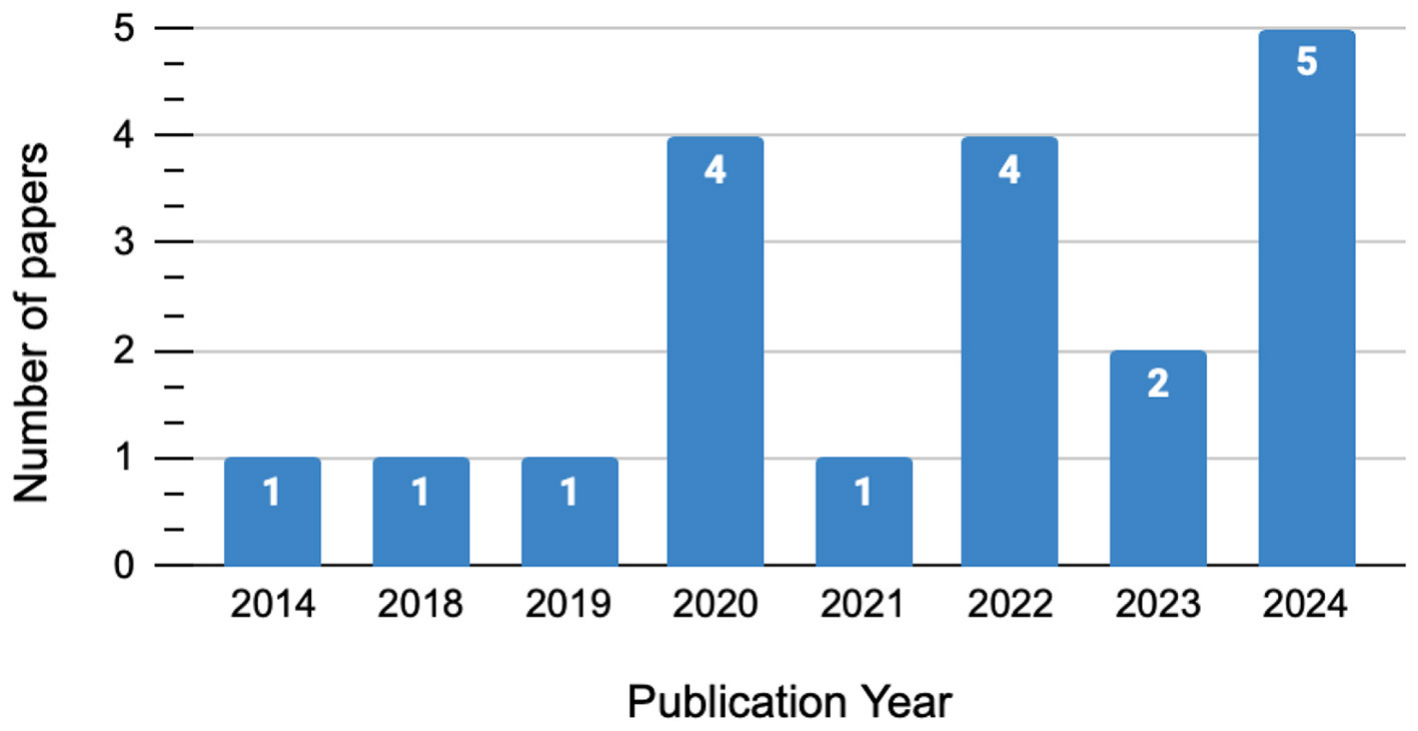
Growth of research publications in the field over the past decade.

The illustrated statistics in Figure 3 show a clear increase in publications over recent years, with a noticeable peak of seven studies between 2023 and 2024. This growing interest suggests that the academic community is placing more focus on this topic.

In contrast, the lower number of publications before 2019 indicates that there may have been a lack of research on this topic, with three studies published. The recent increase reveals a more intensive focus on this field, likely because of the greater relevance and resource allocation in both theoretical and practical contexts.

**Figure 4:**
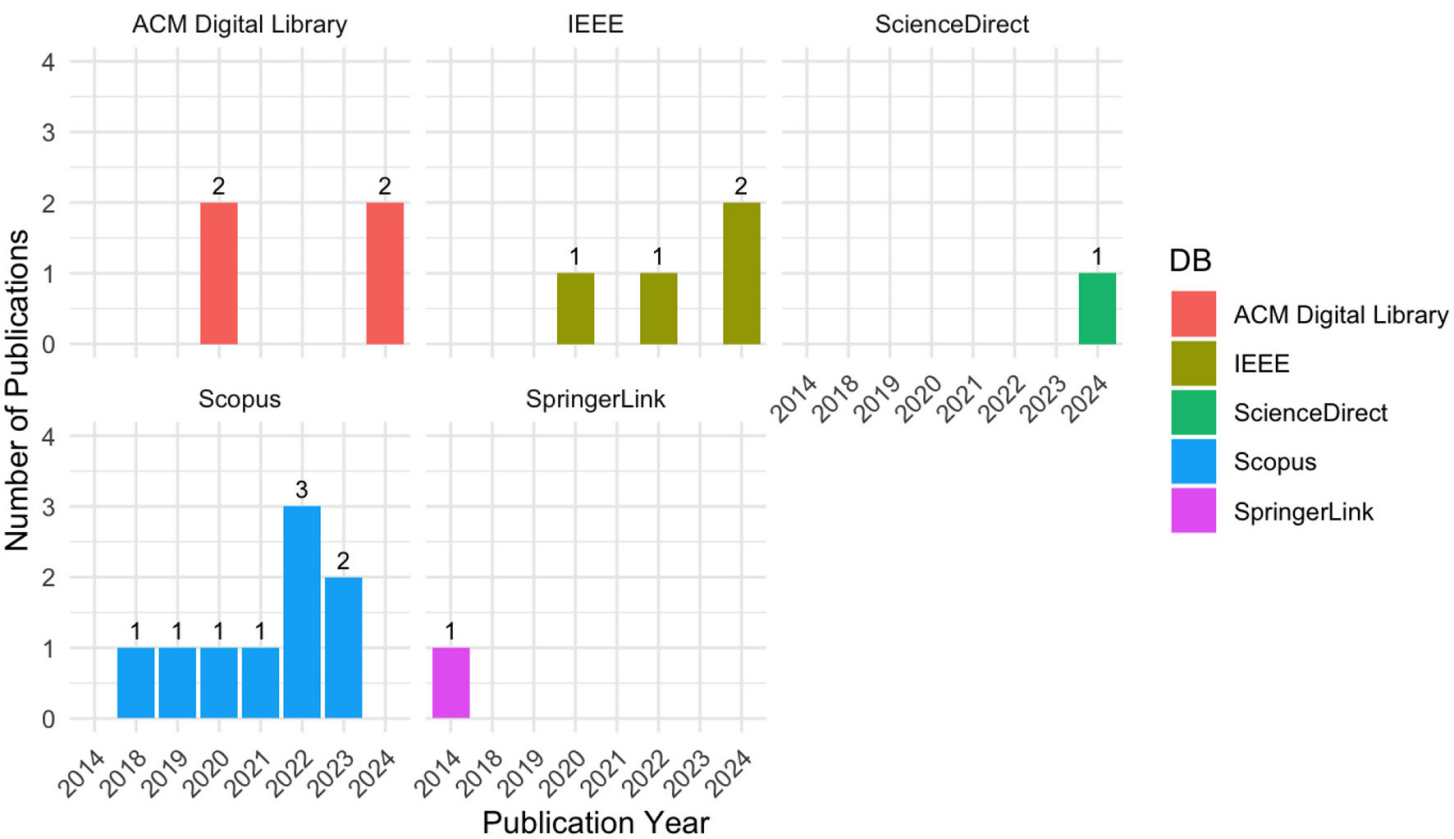
Annual publication trends across scientific databases.

Examining the total number of publications per year by database in Figure 4 reveals that Scopus, ACM Digital Library, and IEEE consistently produce a high volume of publications over years, with recent peaks in 2023 and 2024. This trend indicates that these databases serve as key sources for cutting-edge research as the field evolves. SpringerLink and ScienceDirect also show growth in recent years but with less pronounced volume increases.

**Figure 5:**
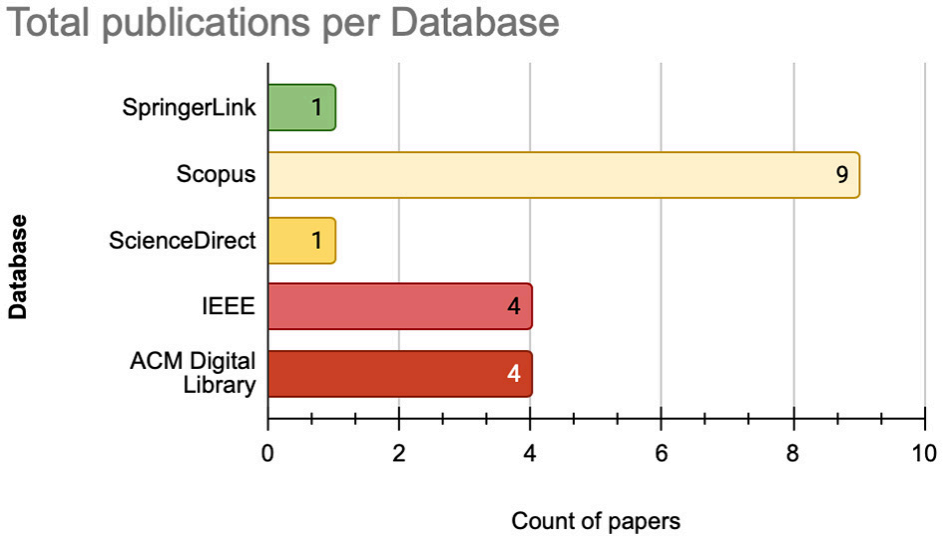
Number of published articles per database.

From the perspective of scientific research databases, as illustrated in Figure 5, SCOPUS stands out as the most prominent database, hosting the largest number of publications with nine publications. Followed by the ACM Digital Library and IEEE Xplore with four publications each. In contrast, SpringerLink and ScienceDirect have a smaller representation, hosting one publications each.

**Figure 6:**
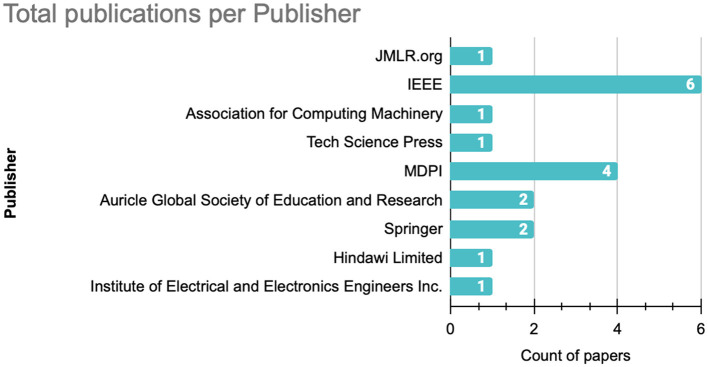
Number of published articles per publisher.

From the publishers perspective, as shown in Figure 6, IEEE and MDPI demonstrate significant involvement in the field, publishing respectively six and four publications. The remaining publications are distributed relatively evenly across other publishers, each contributing with one or two publications.

**Figure 7:**
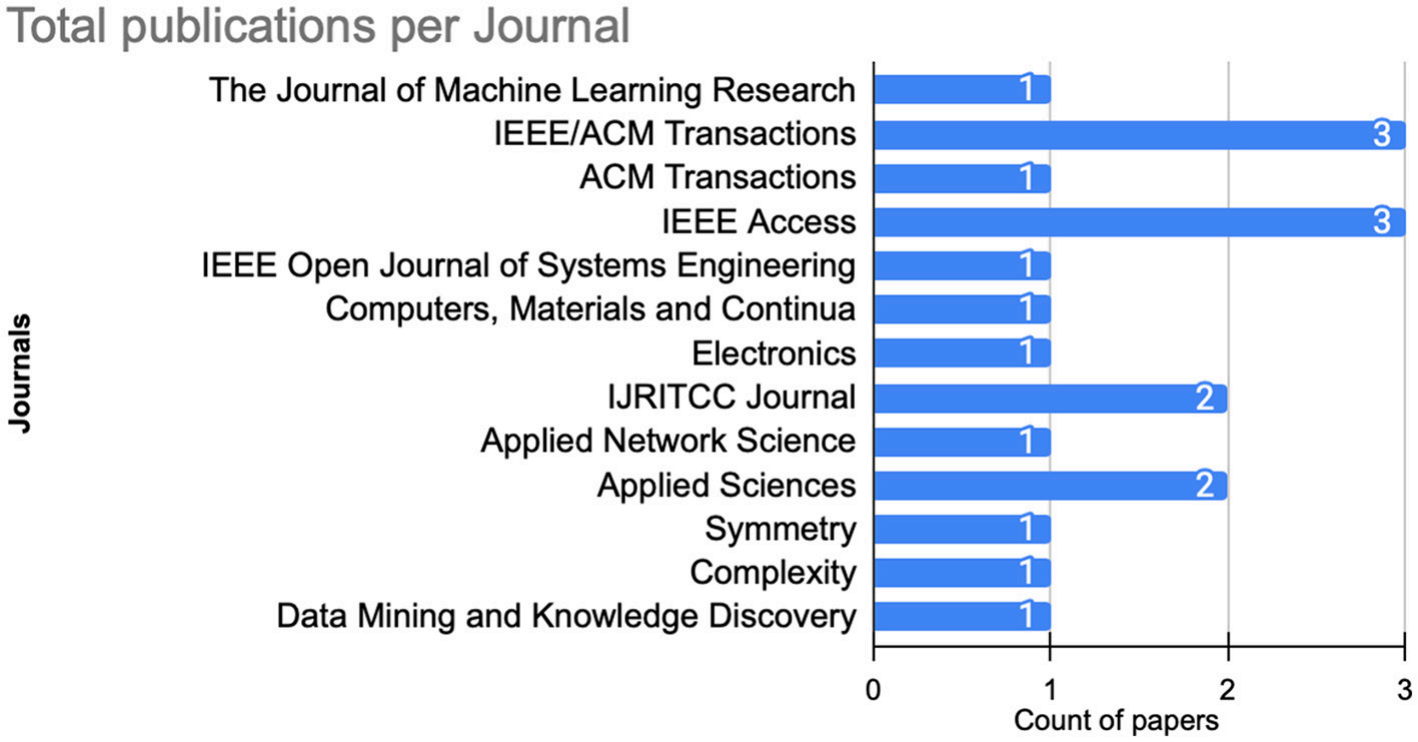
Number of published articles per journal.

Finally, from the journal perspective, as illustrated in Figure 7, IEEE Access and IEEE/ACM Transactions dominate, collectively hosting three publications. Where Applied Sciences and IJEITCC journals host two publications each. The remaining publications are evenly distributed across other journals, with each contributing with one publications.

Our final corpus of 19 studies, as described in Table 4, was selected through iterative screening as described in Section 3.2.3.

**Table 4:** List of final corpus of the 19 selected studies.

**Id**	**Paper**	**Title**
1	Klepper et al. (2024)	Clustering with tangles: algorithmic framework and theoretical guarantees
2	Zhong and Gu (2020)	Predicting local protein 3D structures using clustering deep recurrent neural network
3	Fan et al. (2024)	Behave differently when clustering: a semi-asynchronous federated learning approach for IoT.
4	Karim et al. (2020)	Convolutional embedded networks for population scale clustering and bio-ancestry inferencing
5	Kumar et al. (2024)	Enhanced community detection via convolutional neural network: a modified approach based on MRFasGCN algorithm.
6	Karaa et al. (2024)	A dataset annotation system for snowy weather road surface classification.
7	Bourhim et al. (2022)	A community-driven deep collaborative approach for recommender systems.
8	Wu et al. (2020)	Deep learning techniques for community detection in social networks.
9	Yu et al. (2024)	Social robot detection method with improved graph neural networks.
10	Xia et al. (2023)	Community-enhanced contrastive learning for graph collaborative filtering.
11	Selvakumar and Kathiravan (2023)	Deep learning based Densenet convolution neural network for community detection in online social networks.
12	Jadhav and Burra (2022)	Deep learning in social networks for overlappering community detection.
13	Sobolevsky and Belyi (2022)	Graph neural network inspired algorithm for unsupervised network community detection.
14	Zhao et al. (2022)	A scalable deep network for graph clustering via personalized PageRank.
15	Ferraro et al. (2021)	Deep-learning-based community detection approach on multimedia social networks.
16	Geng et al. (2020)	Network structural transformation-based community detection with autoencoder.
17	Choong et al. (2018)	Variational approach for learning community structures.
18	Levie et al. (2019)	CayleyNets: graph convolutional neural networks with complex rational spectral filters.
19	Choo et al. (2015)	Weakly supervised nonnegative matrix factorization for user-driven clustering.

## 4 Results

### 4.1 RQ1: What are the most commonly used deep learning techniques in community detection?

**Figure 8:**
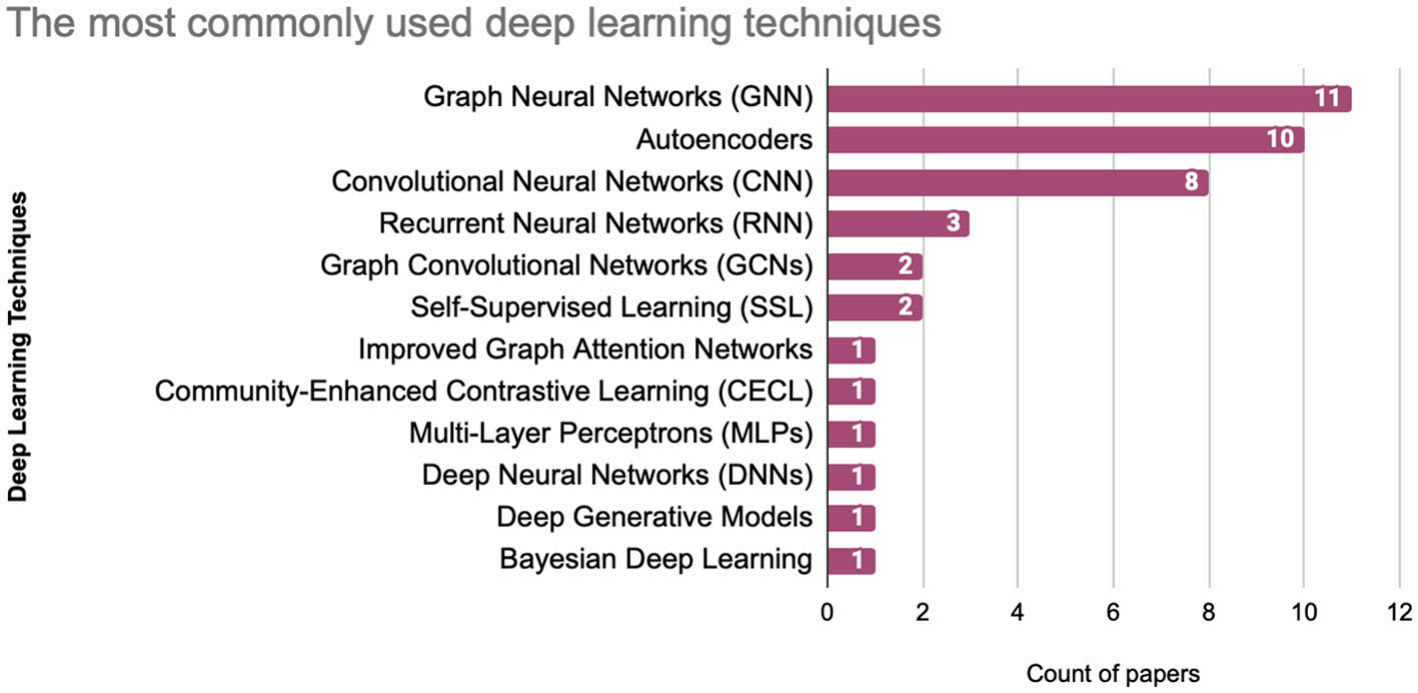
Most commonly used deep learning techniques.

Over the past decade, various deep learning techniques have been employed to address the challenges associated with community detection. Figure 8 highlights the prevalence of these techniques in community detection research. Among them, Graph Neural Networks (GNNs) stand out as the most frequently utilized method, appearing 11 times. Autoencoders follow closely with 10 appearances, and Convolutional Neural Networks (CNNs) with ight appearances.

Recurrent Neural Networks (RNNs) have been employed 3 times, specifically in applications involving temporal graph data. Where the Graph Convolutional Networks (GCNs) and Self-Supervised Learning (SSL) were utilized twice each. Meanwhile, the Specialized methods, including Improved Graph Attention Networks (GANs), Community-Enhanced Contrastive Learning (CECL), and Bayesian Deep Learning appeared only once.

Similarly, foundational architectures such as Multi-Layer Perceptrons (MLPs) and Deep Neural Networks (DNNs) also show limited use, specifically due to their general-purpose nature being less suited for the specialized requirements of graph-based problems.

These findings highlight a strong preference for graph-specific and feature extraction-focused models, underscoring the importance of approaches designed to handle the unique complexities of graph data. This provides valuable guidance for future research, suggesting opportunities to refine existing methods while exploring less common techniques for innovative applications.

### 4.2 RQ2: How are deep learning models for community detection structured and documented in the literature?

**Figure 9:**
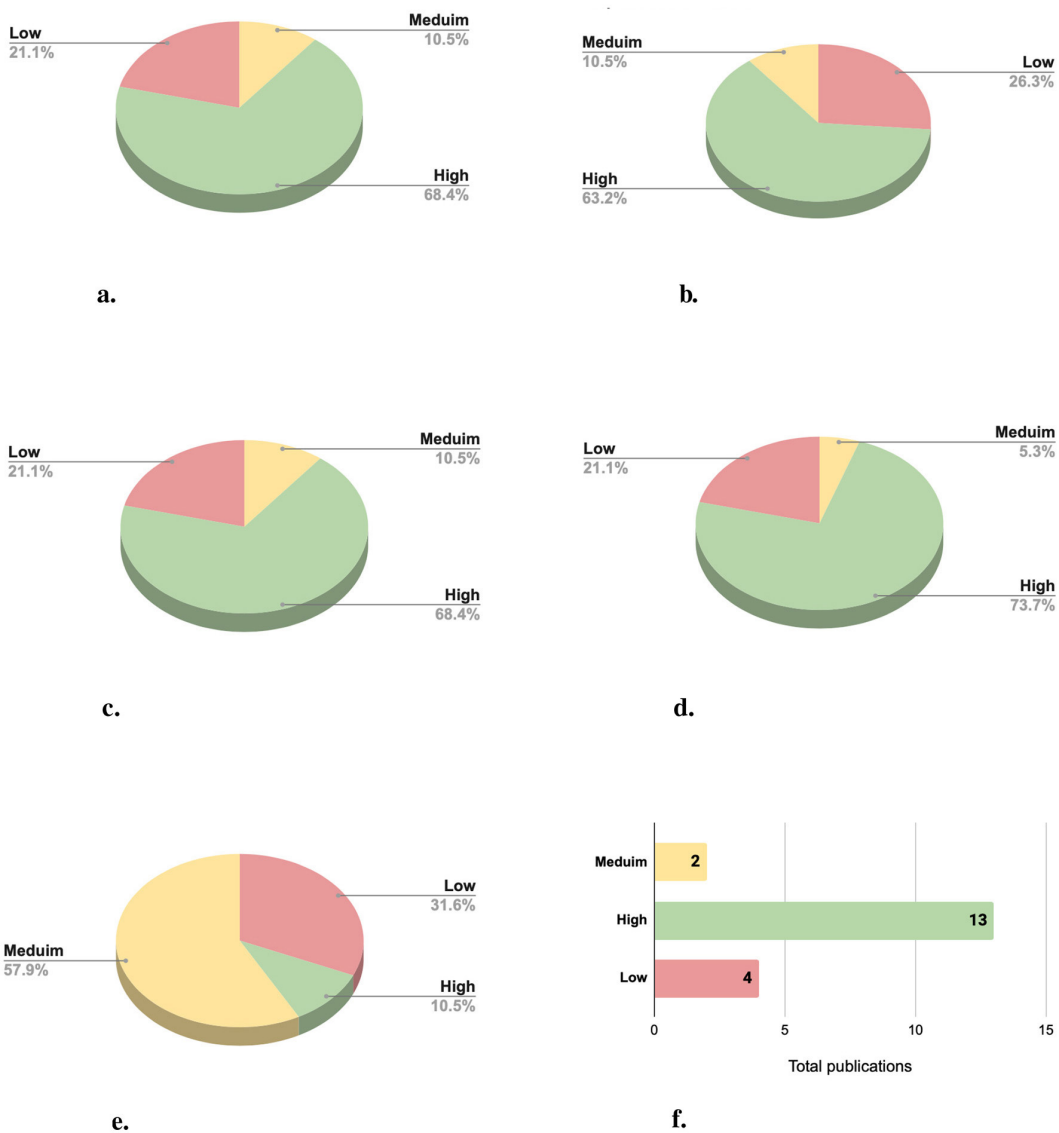
Explicitness of deep learning approaches for community detection across selected publications: **(a)** Model description, **(b)** documentation and input representation, **(c)** training process, **(d)** evaluation design, **(e)** reproducibility, and **(f)** the overall explicitness.

The performed classification system evaluates five key aspects of the selected research papers: (i) model description, (ii) documentation and input representation, (iii) training process, (iv) evaluation design, and (v) reproducibility. Each criterion is assessed on a scale of low, medium, or high, reflecting the depth and the quality of the provided presentation of the deep learning approach for community detection purpose. The Figure 9 describes the explicitness ranking over the five above criteria (Figures 9a–e).

The evaluation of the overall in-depth explicitness across the selected publications reveals significant variability in the level of detail and rigor provided in the research as illustrated in Figure 9f. The distribution of publications into three levels: Low, Medium, and High provides valuable insights into the current state of explicitness in the proposed deep learning techniques applied to community detection.

Among the 19 evaluated publications, the majority of 13 publications (68%) have been classified with high explicitness ratio, reflecting comprehensive and well-documented studies. These publications excelled in providing detailed model descriptions, clear input representations, thorough training process explanations, structured evaluation designs, and reproducible implementation details. Such explicitness indicates a mature understanding of the need for transparency and reproducibility in this domain.

In contrast, low explicitness was observed in four publications (21%). These studies often lacked critical details, such as clear descriptions of the model architecture, preprocessing steps, and reproducibility. The remaining two publications (11%) were classified as Medium, representing a transitional level of explicitness. These categories if studies offered a reasonable level of detail. However, they lacked detail in explaining the input representation or describing the training process.

### 4.3 RQ3: What types of graph or network structures are primarily addressed in deep learning-based community detection?

**Figure 10:**
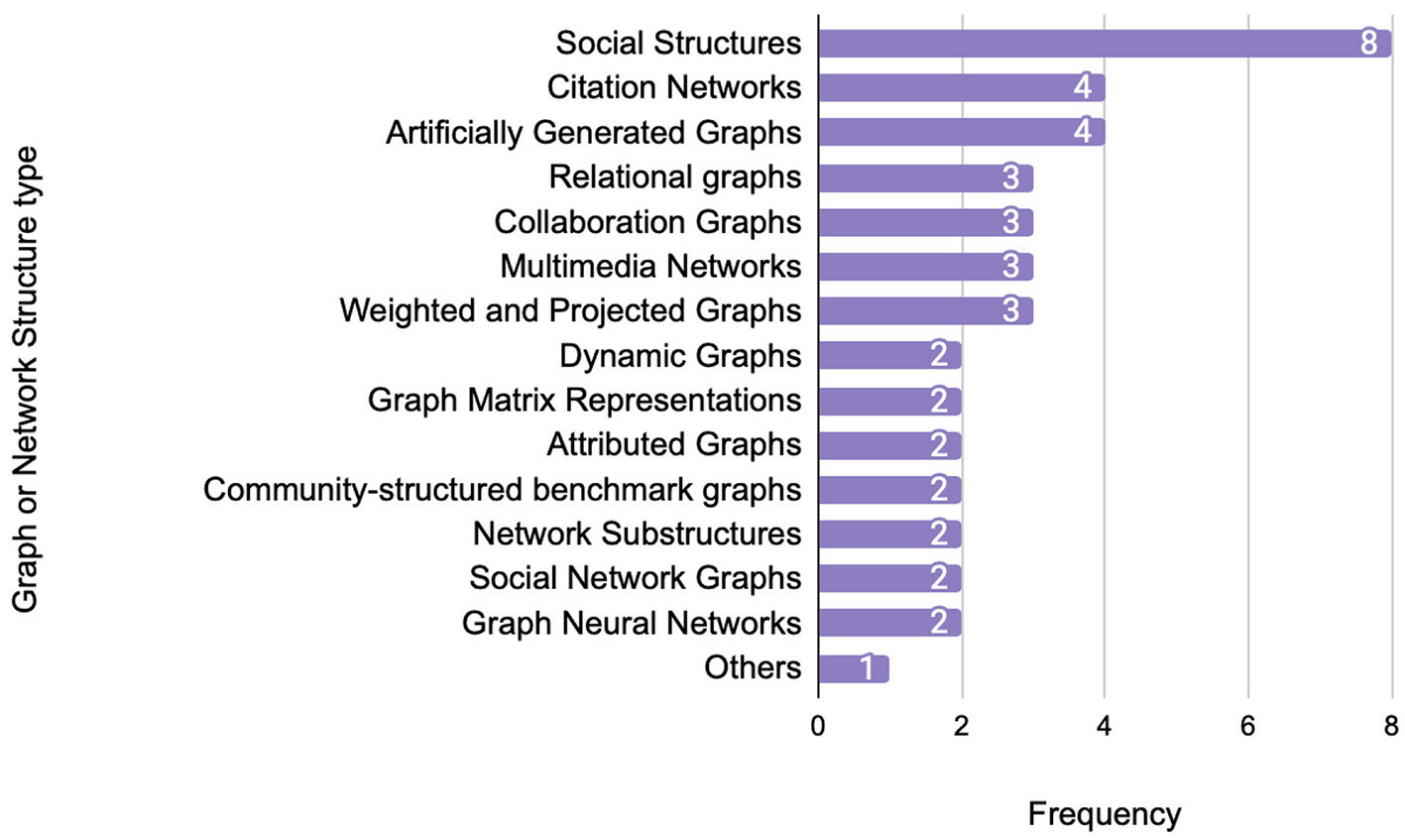
Most commonly used graph or network structures.

Figure 10 highlights the distribution of network structures adopted in the reviewed studies, reflecting the diversity and relevance of graph types for different application domains. Social networks dominate the landscape, appearing in eight studies, highlighting their central role in modeling user interactions and relational behaviors.

Other moderately frequent structures include citation networks and artificially generated graphs (each used in four studies), as well as collaboration, relational, and projected/weighted graphs (three studies each). These graphs support various domains such as academic influence modeling, knowledge graphs, multimedia processing, and user-item interactions.

Less commonly used structures include graph matrix representations, community-structured benchmark graphs, and notably dynamic or temporal graphs, each appearing in only two studies. This suggests that while the dynamic nature of real-world networks (e.g., social or communication platforms) is widely acknowledged, the actual integration of temporal aspects into DL-based community detection remains underdeveloped.

We also observed considerable inconsistency in the way in which few studies that address dynamic networks implemented temporal modeling. Techniques such as snapshot comparison, time windowing, and update mechanisms were used, but with varying assumptions and without standardized evaluation benchmarks. For example, while one model tracked evolving community assignments over discrete intervals, others applied recurrent or sequence-aware architectures without consistent baselines or datasets.

These observations confirm the early stage and fragmented nature of research on dynamic graph community detection using deep learning. The current lack of methodological consistency and the underutilization of standard dynamic benchmarks limit comparability across studies and highlight a clear direction for future investigation. Dedicated efforts to systematically evaluate dynamic graph modeling strategies, supported by unified benchmarks and metrics, are necessary to advance the field.

### 4.4 RQ4: What are the key performance metrics used to evaluate deep learning models for community detection?

**Figure 11:**
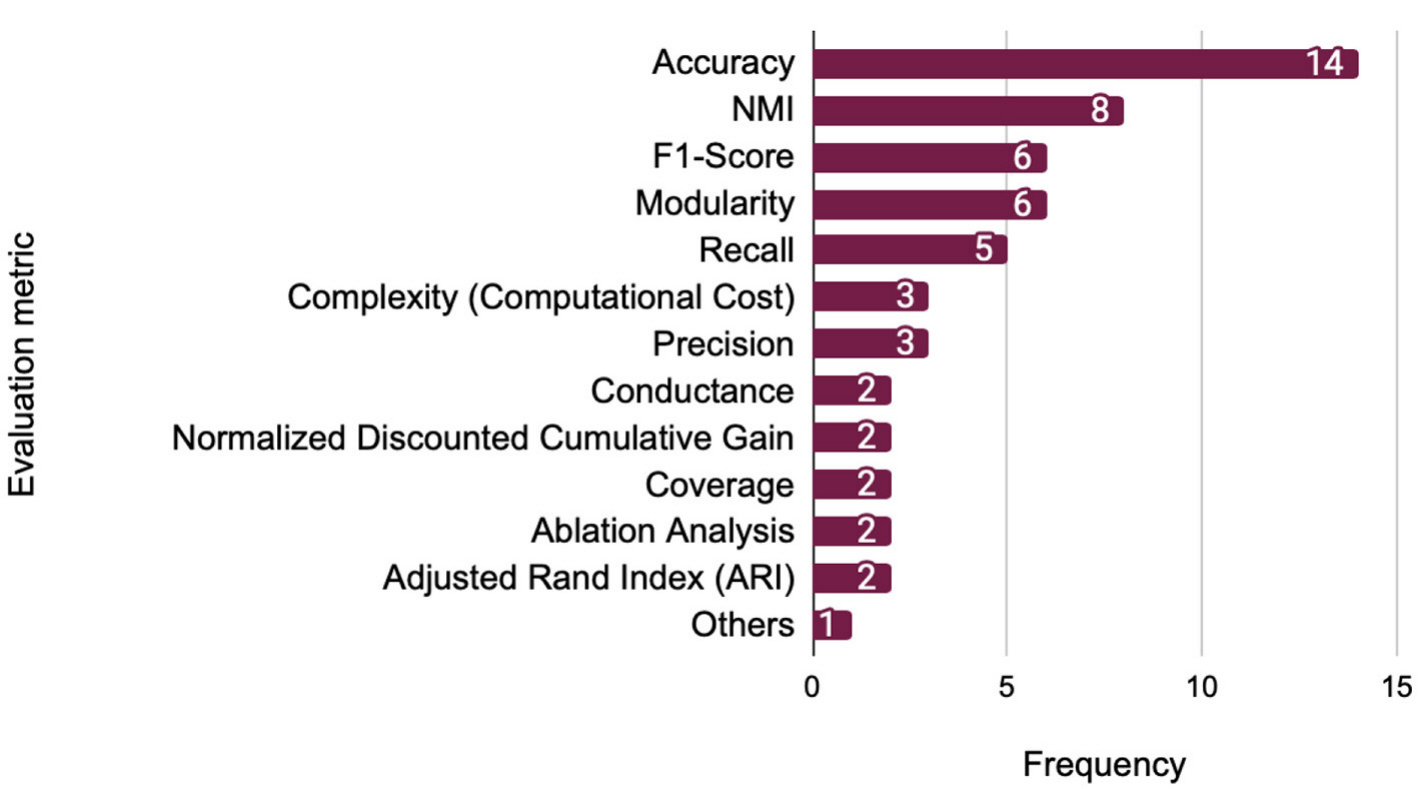
Most commonly used evaluation metrics.

Figure 11 presents the frequency of evaluation metrics utilized in the reviewed studies, highlighting the preferred approaches for assessing graph-based methodologies.

Accuracy is the most commonly used metric (14 studies), reflecting its general applicability in scenarios where ground truth community labels are available. It is especially prevalent in studies using synthetic graphs (e.g., LFR benchmark) or labeled datasets such as DBLP or Amazon, where the prediction of node-community assignments can be directly evaluated.

Normalized Mutual Information (NMI) (eight studies) is a standard measure for comparing the similarity between predicted and ground-truth community structures. It is particularly effective in synthetic settings with well-defined ground truth and in non-overlapping community detection tasks. For example, NMI is commonly used in studies evaluating performance on benchmark graphs like LFR or GN, where the quality of clustering alignment is critical.

F1-Score and Modularity (six studies each) serve different but complementary purposes. F1-Score balances precision and recall, making it suitable for overlapping community detection or multi-label settings where nodes may belong to multiple communities. In contrast, Modularity evaluates the structural cohesiveness of detected communities and is often applied in real-world networks (e.g., citation or social networks) without known ground truth to assess how well the method uncovers densely connected substructures.

Precision (three studies) and recall (five studies) are often reported alongside F1-Score, especially in classification-based or link prediction-inspired community detection tasks. Their use is more prevalent when a fine-grained evaluation of true vs. false positives is required, such as in fraud or anomaly detection applications.

Computational complexity (three studies), while less common, is crucial in large-scale or dynamic graph scenarios. It provides insights into scalability and resource efficiency, especially in works proposing models for real-time community detection.

Other specialized metrics include Conductance useful for evaluating community boundary sharpness in large or hierarchical graphs, which measures the ratio of external to internal edges in communities, and Normalized Discounted Cumulative Gain (NDCG), which is occasionally used in ranking-based evaluations. Ablation Analysis helps assess the individual contribution of model components, especially in multi-module deep learning architectures.

As summarized in Table 5, a limited set of benchmark datasets, such as Cora, DBLP, LFR, and Karate Club, emerge as the most comprehensively evaluated across all core performance metrics (Accuracy, NMI, F1-Score, and Modularity). Their frequent use reflects both their accessibility and their suitability for testing a wide range of deep learning models in community detection. Conversely, several real-world networks (e.g., Facebook, US Airports) and application-specific datasets (e.g., MovieLens, MNIST) appear in fewer studies and are typically evaluated with a narrower set of metrics, often tailored to domain-specific tasks. Notably, Precision and Recall are under-reported across the reviewed studies, underscoring a gap in holistic evaluation. Which can be highlight as a need for more diverse and standardized evaluation protocols that incorporate multiple metrics across both synthetic and real-world datasets.

**Table 5:** Datasets used in model evaluation across different performance metrics.

**Dataset**	**NMI**	**Accuracy**	**F1-score**	**Modularity**	**Recall**
Cora/CORA	Y (3)	Y (4)	Y (3)	Y (3)	–
DBLP	Y (3)	Y (2)	Y (2)	Y (2)	–
LFR/LFR network	Y (3)	Y (2)	Y (2)	Y (2)	–
Karate club/Zachary's karate club	Y (2)	Y (2)	Y (2)	Y (2)	–
GN network	Y (2)	Y (2)	Y (1)	Y (1)	–
Flickr	Y (2)	Y (2)	Y (1)	Y (1)	–
Polblogs/PolBlogs dataset	Y (2)	Y (2)	Y (1)	Y (1)	–
Citeseer/CiteSeer	Y (2)	–	Y (1)	Y (1)	–
Stochastic Block Model (SBM) graphs	Y (2)	–	Y (1)	Y (1)	–
MNIST	–	Y (2)	–	–	–
MovieLens/MovieLens-1M	–	Y (2)	–	–	Y (1)
Citation network	Y (1)	Y (1)	–	–	–
US airports network	Y (1)	–	Y (1)	Y (1)	–
Football	Y (1)	Y (1)	–	–	–
Dolphins	Y (1)	Y (1)	–	–	–
Les Misérables co-occurrence Network	Y (1)	–	Y (1)	Y (1)	–
Facebook	Y (1)	–	–	–	–
PubMed citation network	Y (1)	Y (1)	Y (1)	Y (1)	–
Reuters	Y (1)	Y (1)	–	–	–
ACM	Y (1)	Y (1)	–	–	–
Wiki	Y (1)	–	Y (1)	Y (1)	–
Polbooks networks	Y (1)	Y (1)	–	–	–
Twitter	–	–	Y (1)	Y (1)	–
Kaggle repository datasets	–	Y (1)	–	–	Y (1)

Y indicates the metric is reported; numbers in parentheses indicate the number of occurrences across studies.

In summary, while there is a preference for widely adopted metrics such as Accuracy and NMI, the choice of evaluation metric is often dictated by the graph type, community structure, and the specific research objective.

### 4.5 RQ5: What are the most prominent machine learning and hybrid approaches used alongside deep learning for community detection?

**Figure 12:**
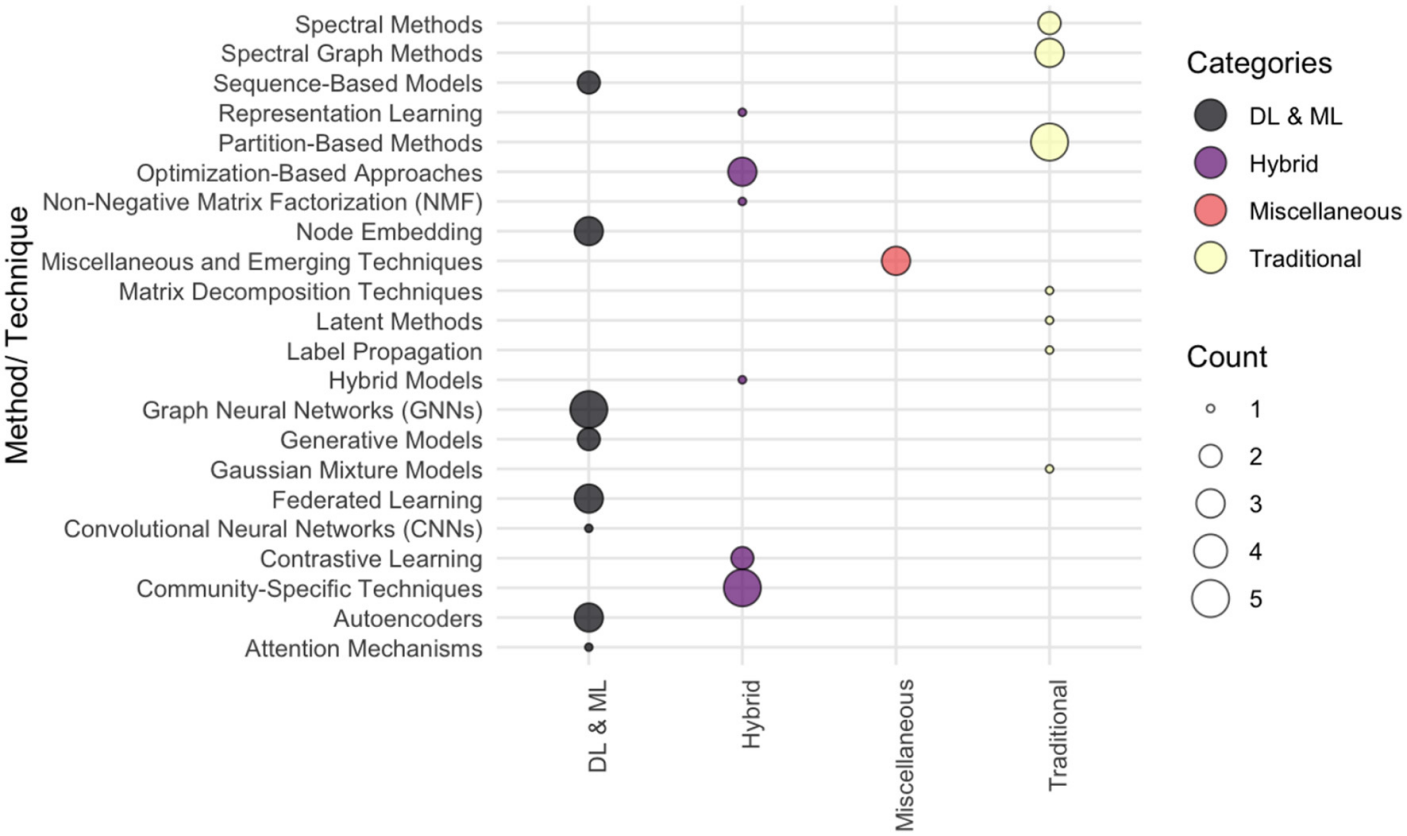
The commonly classification of deep learning techniques used for community detection context.

The Figure 12 illustrates the degree of significance and application of each method used in the surveyed papers. The size of each bubble corresponds to the frequency of the methods within a category, offering a visual representation of their prominence in community detection research.

The findings illustrated in Figure 12 highlight the diverse application of community detection methods across various categories and domains within the surveyed studies. The Deep learning (DL) and Machine learning (ML) techniques dominate the landscape, with Graph Neural Networks (GNNs), Autoencoders, and Node Embedding emerging as the most frequently utilized approaches (Klepper et al., 2024; Bourhim et al., 2022; Jadhav and Burra, 2022). These methods demonstrate their effectiveness in capturing complex patterns and relationships within networks, making them central to advancing the field.

The Hybrid methods, such as Community-Specific Techniques, Contrastive Learning, and Optimization-Based approaches underline the innovative integration of domain-specific knowledge with computational frameworks (Xia et al., 2023; Karaa et al., 2024; Fan et al., 2024). By blending traditional and modern methodologies. Where the Traditional methods, including Partition-Based Methods and Spectral Graph Methods remain integral to the field, especially for benchmark evaluations and use cases where simplicity and interpretability are crucial (Kumar et al., 2024; Geng et al., 2020). The performance of novel techniques can be evaluated using these approaches, which are commonly favored in scenarios that require computational efficiency and straightforward implementation.

The Emerging techniques, such as Generative Models and Federated Learning represent a perspective trend addressing contemporary challenges such as scalability, privacy preservation, and computational constraints (Karim et al., 2020; Zhao et al., 2022; Choo et al., 2015). These methods highlight the ongoing innovation in the field, leading to robust and adaptable solutions in community detection.

Both foundational approaches and cutting-edge innovations are crucial for achieving balanced distribution of community detection methods across these categories. Researchers can improve community detection by incorporating traditional, hybrid, and emerging methods, as well as addressing the diverse needs and challenges of different network types and applications.

### 4.6 RQ6: What datasets are commonly used to train and evaluate deep learning models for community detection?

The surveyed papers reveal the diverse use of datasets in community detection research as summarized in Table 6. Citation and Academic Networks, including CORA^1^ (Sen et al., 2008) with five occurrences, DBLP^2^ (Ley, 2002) with four occurrences, and Citeseer^3^ (Sen et al., 2008) with three occurrences, dominate the field due to their well-structured graph properties, making them ideal for benchmarking graph-based and deep learning techniques. Social and Collaborative Datasets, such as Facebook^4^ (Viswanath et al., 2009), Twitter^5^ (De Choudhury et al., 2010), and Flickr^6^ (Mislove et al., 2008), used widely for studying social and collaborative interactions.

**Table 6:** Datasets classified by category with frequency of occurrence.

**Category**	**Dataset**	**Frequency**
Synthetic and artificial network	Stochastic Block Models (SBM)	4
Synthetic and artificial network	Zachary's Karate Club	3
Synthetic and artificial network	Co-occurrence Networks	2
Citation and academic networks	CORA	5
Citation and academic networks	DBLP	4
Social and collaborative networks	Facebook	3
Social and collaborative networks	Twitter	2
Biological and genomic	1000 Genomes Project (1000GP)	2
Human activity and recognition	UCI-HAR	3
Domain-specific networks	Kaggle repository	3
Domain-specific networks	20 Newsgroups (20News)	2
Synthetic and artificial networks	Artificial Datasets	1

The Graph and Network datasets, like Stochastic Block Models (SBM) (Abbe, 2018) appearing in four occurrences, and Zachary's Karate Club^7^ (Zachary, 1977) in three appearances, provide benchmark graph structures for testing algorithms. Human Activity and Recognition Datasets, including UCI-HAR^8^ and MNIST^9^ showcase the application of graph representations in activity and visual data analysis.

Finally, the Domain-Specific Datasets such as 20 Newsgroups^10^ (Rennie, 2003) and Biological and Genomic Datasets such as 1000 Genomes Project^11^ (Siva, 2008) appear less frequently but highlight the versatility of community detection methods in specialized applications.

### 4.7 RQ7: What tools and frameworks are utilized for implementing deep learning models in community detection?

**Figure 13:**
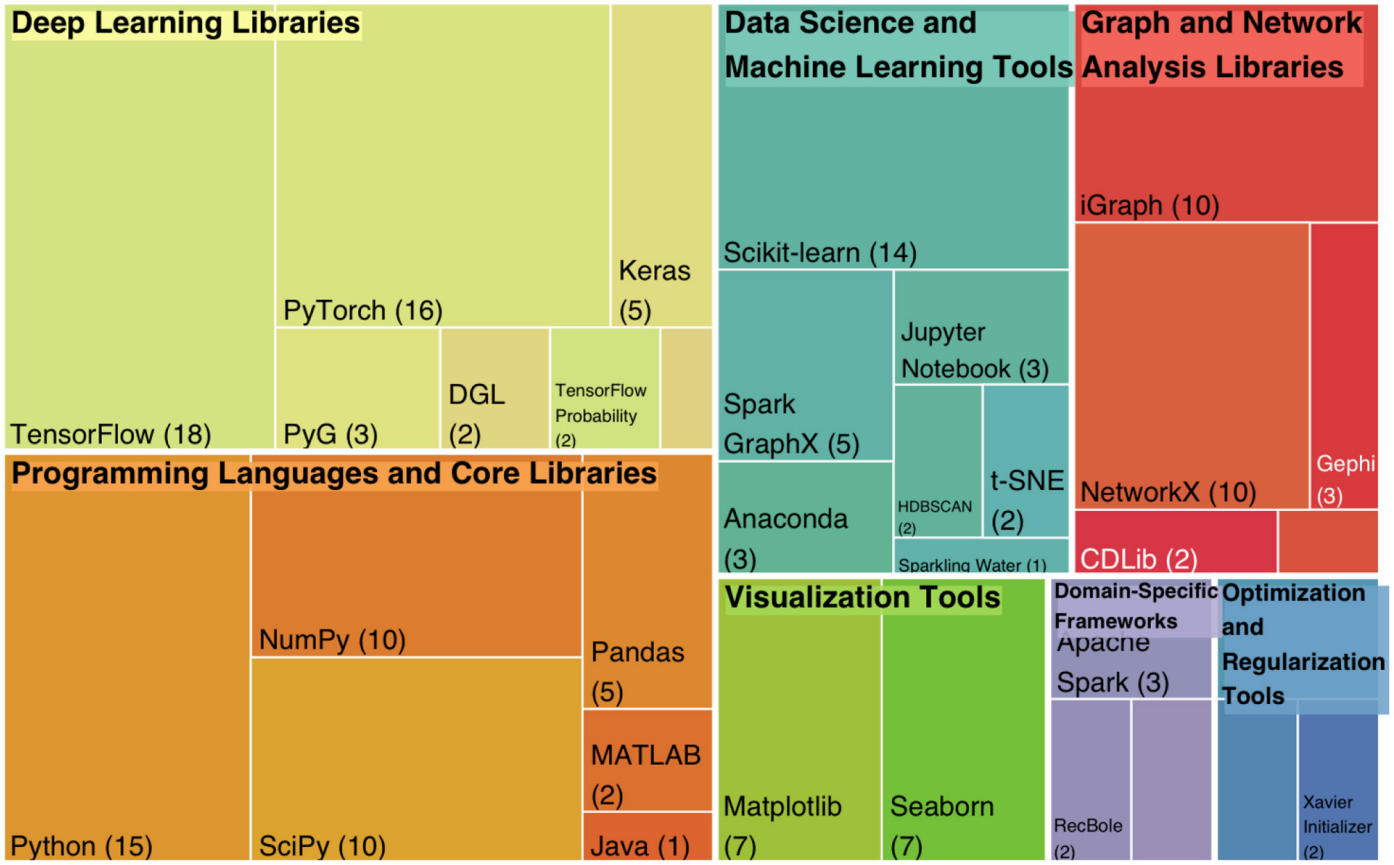
Treemap of the deep learning frameworks usage in community detection research.

The surveyed papers reveal a diverse range of frameworks and tools utilized in community detection research, highlighting their significance across various methodologies. Programming Languages and Core Libraries, particularly Python used in 10 papers, dominate as the primary choice due to their versatility and integration with deep learning and graph analysis libraries. Supporting NumPy and SciPy further enhance computational efficiency and scientific computing capabilities. The accompanying Figure 13 illustrates a treemap representation of the most commonly used frameworks across the surveyed papers, providing a visual summary of their frequency.

Among Deep Learning Libraries, TensorFlow used in 10 papers and PyTorch employed in 11 papers emerge as the most frequently used frameworks, reflecting their widespread adoption for building scalable and flexible neural network models. Keras integrated in 3 papers and specialized libraries like DGL and PyTorch Geometric (PyG) are also noteworthy for their utility in graph-based learning tasks.

Graph and Network Analysis Libraries, such as NetworkX and iGraph used by 6 papers, facilitate robust graph data manipulation and visualization. Scikit-learn employed in nine papers and other Data Science Tools, including Jupyter Notebook and Spark GraphX, play a crucial role in integrating machine learning techniques with graph-based approaches.

### 4.8 RQ8: What are the common challenges and limitations faced in applying deep learning to community detection?

The surveyed papers highlight a variety of challenges and limitations in community detection research, categorized into common themes. These challenges reflect technical, methodological, and application-specific issues that impact the effectiveness and scalability of existing approaches.

- Scalability and computational complexity: scalability to large networks is a recurring limitation across many papers, such as Kumar et al. (2024), Wu et al. (2020), and Selvakumar and Kathiravan (2023), where handling vast datasets results in high computational costs and inefficiencies. Where the research papers, Karim et al. (2020) and Zhao et al. (2022) emphasize the difficulty of managing high-dimensional data, dynamic networks, and evolving relationships.
- Data quality and representation: data sparsity, noise, and imbalanced datasets are frequently cited concerns, as reviewed in Karaa et al. (2024), Xia et al. (2023), and Ferraro et al. (2021). The cold-start problem and noisy features further exacerbate the difficulty of ensuring robust and reliable community detection models.
- Interpretability and parameter sensitivity: papers such as Klepper et al. (2024), Geng et al. (2020), and Choo et al. (2015) note the trade-off between interpretability and performance, sensitivity to hyper-parameters, and dependence on predefined parameters. These issues challenge the generalization and explainability of models in real-world applications.
- Community detection complexity: the complexity of detecting overlapping, fuzzy, or ambiguous communities as highlighted in the papers (Bourhim et al., 2022; Jadhav and Burra, 2022; Wu et al., 2020). This includes challenges in balancing local and global network properties and resolving ambiguities in community definitions.
- Evaluation challenges: a lack of benchmark datasets, ground truth ambiguity, and limitations of existing evaluation metrics are underscored in papers (Kumar et al., 2024; Karaa et al., 2024; Sobolevsky and Belyi, 2022). These challenges make it difficult to validate and compare the effectiveness of different models.
- Dynamic and temporal networks: papers such as Yu et al. (2024) and Ferraro et al. (2021) highlight difficulties in modeling temporal and dynamic networks, where relationships and structures evolve over time, adding complexity to feature integration and model consistency.
- Model Design and over-fitting: over-fitting on small datasets and balancing model complexity are notable concerns in papers (Levie et al., 2019; Selvakumar and Kathiravan, 2023), where improper generalization can compromise model performance in diverse datasets.

As shown in Table 7, Graph Neural Networks (GNNs) are the most frequently associated with multiple challenges, notably scalability, sparsity, overlapping communities, parameter sensitivity, and interpretability. MLP-based models also show limitations in handling dynamic and high-dimensional networks, while autoencoders and hybrid architectures are primarily linked to issues of interpretability and parameter tuning. RNNs/LSTMs are uniquely suited to temporal dynamics but lack broader adaptability to structural graph complexities. Meanwhile, contrastive and generative models appear underrepresented in challenge mappings, highlighting a gap in systematic evaluation. These findings suggest the need for more adaptable and interpretable models, especially those capable of addressing sparsity, temporal variability, and the evolving structure of real-world networks.

**Table 7:** Mapping of deep learning model types to challenges and limitations categories.

**DL model type/associated challenges**	**A**	**B**	**C**	**D**	**E**	**F**	**G**	**H**
Graph Neural Networks (GNNs)	Y	Y	Y	Y	Y	–	–	–
Recurrent Neural Networks (RNNs) or LSTMs	–	–	–	–	–	Y	–	–
Convolutional Neural Networks (CNN)/spectral CNN	Y	–	–	–	–	–	–	Y
Autoencoders (AE, CAE, Denoising AE)	–	Y	–	Y	Y	–	–	–
Graph Convolutional Neural Networks (GCN)/Improved Graph Attention Networks (GATs)/Recurrent Graph Neural Networks	Y	–	–	–	–	–	–	–
Hybrid Models (e.g., GNN+AE, GCN+CNN)	–	–	–	Y	Y	–	–	–
Deep Generative models/Bayesian deep learning	–	–	–	–	–	–	–	–
Community-Enhanced Contrastive Learning (CECL) Models	–	Y	–	–	–	–	–	–
MLP-based/Dense DNNs	Y	–	Y	–	–	–	Y	Y

Challenges—A: scalability and complexity, B: sparsity, C: overlapping communities, D: parameter sensitivity, E: interpretability, F: temporal dynamics, G: handling dynamic graphs, H: high-dimensional network features.

The limitations identified in the surveyed studies collectively emphasize the need for scalable, interpretable, and robust community detection models. These models must be capable of effectively handling noisy, dynamic, and high-dimensional data while maintaining their applicability across diverse real-world scenarios. The recurring challenges, including the trade-offs between computational efficiency, model accuracy, and adaptability, underscore the complexity of achieving a holistic solution.

### 4.9 RQ9: What are the emerging trends in the application of deep learning to community detection?

The Table 8 presents the emerging trends identified in the reviewed studies within this SLR. These emerging trends demonstrate the ongoing evolution of deep learning applications within community detection topic. The performed analysis reveals a wide range of advancements, including innovative methodologies, the incorporation of novel techniques, and customized adaptations for specific applications.

**Table 8:** The identified emerging trends in the surveyed papers within the current SLR.

**Emerging trends**	**Total papers**	**Papers**
Graph Neural Networks (GNNs) and related techniques	3	Klepper et al., 2024; Bourhim et al., 2022; Sobolevsky and Belyi, 2022
Hybrid approaches	3	Zhong and Gu, 2020; Karaa et al., 2024; Jadhav and Burra, 2022
Self-supervised and semi-supervised learning	3	Klepper et al., 2024; Xia et al., 2023; Ferraro et al., 2021
Temporal and dynamic networks	3	Wu et al., 2020; Sobolevsky and Belyi, 2022; Yu et al., 2024
Dimensionality reduction and feature integration	3	Karim et al., 2020; Jadhav and Burra, 2022; Karaa et al., 2024
Scalability and efficiency	4	Zhao et al., 2022; Zhong and Gu, 2020; Levie et al., 2019; Sobolevsky and Belyi, 2022
Domain-specific applications	2	Yu et al., 2024; Choo et al., 2015

#### 4.9.1 Graph Neural Networks (GNNs) and related techniques

Graph Neural Networks (GNNs) have emerged as a prominent focus in the reviewed studies (Klepper et al., 2024; Bourhim et al., 2022; Sobolevsky and Belyi, 2022), reflecting their extensive applicability and versatility. Recent advancements have expanded their capabilities, including the development of Graph Attention Networks (GATs) handling effectively sparse and large-scale graphs. Simplified GNN architectures have been introduced to improve scalability, making them more suitable for complex and resource-intensive tasks. In addition, GNNs have been integrated with multimodal and heterogeneous data representations, extending their scope to diverse applications.

Methods such as personalized PageRank and self-supervised learning further enhance their adaptability, particularly in dynamic and evolving network environments, which improved their ability to capture complex relationships and changes over time.

#### 4.9.2 Hybrid approaches

Hybrid methods combining traditional and deep learning techniques are prevalent across multiple studies (Zhong and Gu, 2020; Karaa et al., 2024; Jadhav and Burra, 2022). These include clustering-based optimization, fusion of static and dynamic features, and integration of graph-based methods with autoencoder or convolutional neural network (CNN) frameworks. Such approaches improve scalability, efficiency, and the ability to capture both local and global network properties.

#### 4.9.3 Self-supervised and semi-supervised learning

The growing use of self-supervised learning (Klepper et al., 2024; Xia et al., 2023) and semi-supervised learning (Ferraro et al., 2021) addresses challenges in labeled data scarcity. These methods leverage graph structure and node features to learn representations without extensive manual labeling, making them ideal for large-scale, heterogeneous networks.

#### 4.9.4 Temporal and dynamic networks

The integration of temporal analysis is emphasized in studies like Wu et al. (2020), Sobolevsky and Belyi (2022), and Yu et al. (2024), where dynamic graph modeling is combined with static features to analyze evolving community structures. The fusion of temporal and static features enables real-time detection and predictive applications, crucial for applications like social network analysis and behavioral studies.

#### 4.9.5 Dimensionality reduction and feature integration

Techniques like autoencoders for dimensionality reduction (Karim et al., 2020; Jadhav and Burra, 2022) and latent space representations (Karaa et al., 2024) are widely adopted to manage high-dimensional graph data. Integration of node attributes, topological features, and advanced embedding techniques helps to improve the interpretability and performance of community detection algorithms.

#### 4.9.6 Scalability and efficiency

Scalability remains a critical focus, with efforts to develop scalable GNNs (Zhao et al., 2022) and parallelization techniques (Zhong and Gu, 2020) to handle large datasets efficiently. These methods are complemented by advancements in computational techniques, such as spectral filtering innovations and optimization approaches (Levie et al., 2019; Sobolevsky and Belyi, 2022).

#### 4.9.7 Domain-specific applications

The surveyed papers highlight the customization of DL models for specific domains, such as real-world networks, multi-modal datasets, and heterogeneous networks (Yu et al., 2024; Choo et al., 2015). Domain-specific customization includes addressing class imbalance with weighted loss functions, incorporating prior knowledge, and ensuring flexibility to handle noisy and partial data.

The emerging trends in deep learning for community detection underscore the increasing sophistication and adaptability of the field. The integration of GNNs, hybrid approaches, and self-supervised learning with scalable and domain-specific techniques marks a transformative phase in research. These innovations not only address longstanding challenges like scalability and data sparsity but also open new frontiers for dynamic, real-time, and multimodal applications. This synthesis of trends provides a roadmap for future research, bridging the gap between theoretical advancements and practical implementations in diverse domains.

### 4.10 RQ10: How do the domains or types of networks influence the effectiveness of deep learning in community detection?

The effectiveness of deep learning (DL) in community detection is profoundly influenced by the domain and network type. Networks with hierarchical or well-defined structures, such as social and biological networks, align well with DL approaches due to their modularity and structural coherence. In contrast, sparse, noisy, or dynamic networks introduce challenges requiring tailored pre-processing, parameter tuning, and noise-handling techniques as highlighted in Klepper et al. (2024), Karaa et al. (2024), and Xia et al. (2023).

Domain-specific characteristics play a crucial role in shaping DL methodologies. Biological and genomic networks demand models capable of handling high dimensionality, noise, and domain-specific requirements like interpretability as seen in Karim et al. (2020), and Zhong and Gu (2020), while dynamic and temporal networks necessitate approaches that integrate static and evolving features (Yu et al., 2024; Sobolevsky and Belyi, 2022). Heterogeneous networks further challenge models to reconcile diverse structural and nodal features such as those described in Choong et al. (2018) and Bourhim et al. (2022), with graph neural networks (GNNs) and hybrid methods demonstrating flexibility in extracting both global and local patterns.

The type of network (real-world vs. synthetic; weighted vs. unweighted; or homogeneous vs. heterogeneous) also dictates the choice of DL techniques (Selvakumar and Kathiravan, 2023; Ferraro et al., 2021; Zhao et al., 2022; Levie et al., 2019). Factors like node attributes, graph density, and connectivity significantly impact model performance, while scalability remains a persistent challenge in large-scale networks. These findings underscore the importance of aligning DL models with the specific characteristics of the domain to maximize their effectiveness in community detection as noted in Wu et al. (2020) and Jadhav and Burra (2022).

The methodological choices in deep learning-based community detection are not only influenced by network structures but also by the specific application domains in which these networks originate. While our initial analysis linked network types (e.g., dynamic, hierarchical, sparse) to deep learning effectiveness, further refinement is needed to explicitly assess how domain-specific factors shape methodological decisions. For instance, biological networks require models capable of handling high-dimensional and noisy data with domain-specific constraints such as interpretability. Conversely, social interaction networks emphasize the need for dynamic representations that evolve with user behavior. However, our review found that the majority of surveyed studies focused on network type rather than explicitly addressing domain-related constraints in their methodological designs. Future research should systematically distinguish between the impact of application domains and network types to provide a clearer understanding of how these factors influence deep learning model selection, evaluation strategies, and scalability considerations.

## 5 Discussion

This SLR synthesizes the findings from 19 selected studies to examine the application of DL techniques to community detection in complex networks. The analysis is structured across several dimensions, including model types, dataset choices, implementation practices, and methodological trends. In addition to highlighting advances, this section also discusses unresolved challenges and outlines future research directions.

### 5.1 Model trends and methodological shifts

A significant methodological shift is observed from traditional clustering techniques toward deep learning-driven approaches. Of the reviewed studies, 40% employ pure DL or ML-based models, while 26% adopt hybrid architectures that combine deep learning with classical clustering frameworks. This evolution reflects a growing recognition of DL's ability to capture non-linear, high-dimensional relationships that conventional methods may overlook.

Graph Neural Networks (GNNs) have emerged as the predominant model class, offering advantages in learning from both graph topology and node attributes. GNNs are frequently employed for their scalability to large graphs and ability to generalize across network structures. Autoencoders and Generative Adversarial Networks (GANs) are also common, primarily used for dimensionality reduction and representation learning (Klepper et al., 2024; Bourhim et al., 2022; Jadhav and Burra, 2022). Hybrid models—such as combinations of GNNs with clustering algorithms—demonstrate promising adaptability by bridging structural graph learning with interpretable cluster formation (Zhong and Gu, 2020; Xia et al., 2023; Sobolevsky and Belyi, 2022). Nevertheless, traditional algorithms like K-means and spectral clustering remain present, often used as baseline methods (Klepper et al., 2024; Kumar et al., 2024).

### 5.2 Dataset usage and influence

Dataset selection plays a crucial role in model evaluation and performance. Structured citation networks such as CORA and DBLP are among the most frequently used, offering controlled environments for benchmarking due to their labeled communities and clean structure. In contrast, social networks (e.g., Facebook, Twitter) highlight interaction dynamics and require models capable of handling noisy, sparse, and potentially overlapping communities. The use of artificially generated graphs supports controlled experimentation, while domain-specific datasets (e.g., multimodal, recommender systems) test model robustness under diverse graph conditions (Bourhim et al., 2022; Yu et al., 2024; Selvakumar and Kathiravan, 2023).

However, dynamic and temporal networks remain underexplored. Only five of the reviewed studies addressed time-evolving graphs, with limited consistency in methodology or benchmarks. As highlighted in Section 4.3, this points to a critical gap in the literature and the need for standardized approaches in modeling network dynamics.

### 5.3 Tools and frameworks

From an implementation perspective, Python is the dominant programming language, with TensorFlow and PyTorch as the leading DL frameworks. Graph-specific libraries like NetworkX and iGraph are commonly used for preprocessing and visualization, while domain-specific toolkits such as RecBole have gained traction in recommendation-based community detection scenarios (Klepper et al., 2024; Choong et al., 2018).

### 5.4 Emerging trends

Several promising trends are observed. The adoption of self-supervised and semi-supervised learning techniques is growing, helping to address data scarcity and improve generalization across different graph domains (Klepper et al., 2024; Xia et al., 2023; Zhao et al., 2022). Furthermore, there is increasing interest in modeling dynamic communities, though this remains an emerging area with considerable methodological variation (Wu et al., 2020; Sobolevsky and Belyi, 2022; Levie et al., 2019). Hybrid models continue to attract attention for their ability to integrate local and global perspectives, especially when combining DL-based representation learning with traditional clustering strategies.

Despite recent advancements, deep learning-based community detection continues to face several critical challenges. These include scalability limitations in handling large-scale and dynamic networks (Kumar et al., 2024; Jadhav and Burra, 2022; Sobolevsky and Belyi, 2022), data quality issues such as sparsity and noise (Klepper et al., 2024; Karaa et al., 2024; Xia et al., 2023), and the lack of interpretability inherent to complex model architectures (Klepper et al., 2024; Geng et al., 2020; Choo et al., 2015). Furthermore, the absence of standardized benchmarks and evaluation metrics complicates reproducibility and fair comparison across studies (Bourhim et al., 2022; Sobolevsky and Belyi, 2022; Choong et al., 2018), while generalization across diverse network types remains limited (Levie et al., 2019; Selvakumar and Kathiravan, 2023). These issues underscore the need for more scalable, robust, and transparent approaches. The following section discusses these limitations in greater detail and outlines key directions for future research.

## 6 Limitations and future work

To ensure the replicability of this systematic literature review (SLR), we documented the search terms, inclusion/exclusion criteria, and screening process in detail, as outlined in Section 3.2.3.

### 6.1 Limitations of the review methodology

A key limitation of this study lies in the constraints imposed by the selected search terms and databases, which may have excluded relevant works outside the predefined scope. Additionally, inclusion/exclusion criteria–such as limiting studies to peer-reviewed journal articles in English between 2014 and 2024–ensured a high-quality dataset but potentially excluded valuable conference papers, preprints, and non-English research contributions. Although a systematic search approach using well-constructed keywords and manual verification was applied to mitigate these concerns, emerging trends and ongoing developments in deep learning for community detection may not be fully captured (Kumar et al., 2024; Sobolevsky and Belyi, 2022).

While this systematic review provides valuable insights into deep learning techniques for community detection, certain limitations should be acknowledged. One key limitation is the lack of a dedicated methodological analysis focusing on the influence of application domains. Although we examined the impact of network types on deep learning effectiveness, we did not systematically differentiate between network structure and the specific domain constraints that shape methodological choices. For instance, while biological networks demand models that prioritize interpretability and high-dimensional data processing, social and dynamic networks require adaptive architectures that accommodate evolving interactions. Our findings primarily linked application domains to network types but did not isolate domain-specific methodological adaptations. Addressing this gap requires a structured analysis that explicitly categorizes networks by domain and examines how domain-specific challenges influence deep learning model design, evaluation metrics, and scalability. Future research should explore this distinction to provide a more comprehensive understanding of how deep learning methodologies can be optimized for different application areas.

### 6.2 Limitations of the reviewed studies

From a methodological perspective, our review identified several critical limitations in the surveyed studies that impact the generalizability and effectiveness of deep learning techniques in community detection:

- Reproducibility and implementation gaps—Only 42% of the reviewed studies provided full implementation details, with just Klepper et al. (2024) and Wu et al. (2020) offering containerized solutions. The lack of open-source code and standardized experimental setups limits the ability to replicate findings and compare approaches effectively.
- Scalability constraints—While 73% of studies focused on small graphs (< 100K nodes), only Levie et al. (2019) evaluated billion-edge networks. The limited examination of large-scale and high-dimensional data suggests that scalability remains a significant challenge for deep learning-based community detection methods.
- Benchmarking and evaluation inconsistencies—Despite the diversity of proposed models, 79% of studies used three or fewer baseline methods, and none incorporated recent graph transformers for comparison. Moreover, 89% mixed modularity with clustering metrics without normalization, leading to inconsistencies in performance evaluation.
- Adaptability to real-world networks—Many studies focused on static networks, neglecting the complexities of dynamic and evolving relationships found in social and biological networks. Only five studies explicitly tested models on temporal graphs, and their methodologies varied significantly in terms of time-window selection and update mechanisms.
- Interpretability vs. performance trade-off—The surveyed deep learning models often function as “black boxes,” making it difficult to interpret their decisions. Hybrid models that combine traditional clustering with deep learning offer improved flexibility but further obscure explainability, complicating their real-world adoption (Zhong and Gu, 2020; Sobolevsky and Belyi, 2022).
- Data limitations—Sparse, noisy, and imbalanced datasets hinder model robustness, with non-independent and identically distributed (non-IID) data further complicating distributed learning approaches (Fan et al., 2024; Xia et al., 2023). These issues increase the risk of overfitting and reduce generalization to unseen network structures.

### 6.3 Future research directions

Addressing these limitations requires targeted efforts to enhance scalability, benchmarking consistency, and model interpretability. In particular, future research should consider the evolving complexity of real-world networks and the increasing demand for dynamic adaptability. Priority areas include:

- Developing more efficient and scalable architectures capable of handling billion-edge networks.
- Enhancing standardized evaluation metrics and benchmark datasets to ensure comparability across studies.
- Improving model transparency and interpretability, particularly for hybrid approaches.
- Exploring domain-specific methodological adaptations, explicitly differentiating network types from application domains.
- Advancing deep learning frameworks for dynamic and temporal community detection, including the use of temporal GNNs, continuous learning strategies, and robust modeling of evolving graph structures.

## 7 Conclusion

This systematic literature review (SLR) analyzes 19 studies and addresses 10 critical research questions to clarify the contribution of deep learning (DL) techniques in community detection within social networks. By examining diverse approaches, datasets, frameworks, and applications, the review captures the dynamic evolution of this field, and highlights its growing impact.

The SLR reveals that DL methods have impacted the community detection topic by addressing the challenges created by the complex, large-scale, and dynamic network structures. Techniques such as Graph Neural Networks (GNNs), Autoencoders dominate the landscape, accounting around 40% of the surveyed approaches. These methods excel in capturing intricate patterns, modeling structural relationships, and enabling scalability. Hybrid approaches, integrating traditional methods with modern DL architectures demonstrate a remarkable flexibility and adaptability. The traditional techniques, including Spectral Clustering and K-Means remain relevant as comparative benchmarks. However, their limitations in handling large, heterogeneous, or dynamic networks have turned to more advanced techniques.

The effectiveness of deep learning (DL) methods is also determined by the choice of datasets with regards to network characteristics. Citation networks, including CORA and DBLP, are frequently utilized datasets for evaluation in the surveyed studies, which underscores the significance of adjusting models to the specific properties of networks, which enhance the applicability and performance of DL techniques in diverse network contexts.

From a framework perspective, deep learning frameworks such as TensorFlow, PyTorch, and Python describes the technological choice of researchers. While the choice of domain-specific libraries and tools such as RecBole, NetworkX and iGraph reflects the increasing focus on scalability, domain customization, and practical implementation, which enable researchers to adapt solutions to the unique challenges posed by diverse network structures and applications.

Emerging trends such as self-supervised learning, dynamic network modeling, and federated learning reflect the ongoing innovation in the field of community detection, addressing the need for generalization across domains, and critical challenges such as data scarcity, privacy preservation. Nevertheless, persistent challenges remain, including scalability for large networks, interpretability of complex models, and the lack of standardized evaluation metrics and benchmarks.

This review underscores the transformative potential of deep learning (DL) in advancing the field of community detection. It highlights the progress and maturity that have been achieved to date while identifying essential areas for future exploration, like heterogeneous networks, dynamic community detection, and cross-domain applications. By addressing these challenges and tackling the advantage of emerging trends, the community detection will gain in robustness, scalability, and interpretability of solutions capable of handling the growing complexity of network environments.

Another promising research avenue lies in the integration of Granular-Ball Computing (GBC) into graph-based deep learning models (Yang et al., 2025a,b). Originally proposed to enhance generality and efficiency through adaptive neighborhood construction, GBC replaces point-wise representations with granular-balls, enabling coarse-grained abstraction while preserving local structural fidelity. Recent advancements, such as the introduction of fuzzy granular-ball rough sets for three-way decisions (3WD-FGBRS) and the robust three-way classifier based on shadowed granular-balls (3WC-SGBs), have demonstrated the potential of GBC in handling uncertainty and imprecision in complex decision-making tasks.

These GBC-based models excel in uncertainty-aware classification by partitioning data into core, important, and unessential regions, and by optimizing multi-granularity spaces without relying on subjectively defined risk parameters. Although such innovations have not yet been explored within the context of graph neural networks (GNNs) or community detection, their interpretability, robustness, and adaptability to ambiguous data make them highly relevant. Future research should investigate the integration of GBC principles into GNN architectures for community detection–particularly in noisy, dynamic, or partially labeled networks–to develop globally informed, semantically grounded, and uncertainty-resilient models. Such hybrid approaches may unlock new capabilities for learning from complex and evolving graph structures.

In summary, the integration of deep learning techniques into community detection field is a significant transformation that presents unmatched opportunities for innovation and practical applications. Researchers can use DL to analyze and understand complex network systems in various domains, by enhancing methodologies and evaluation frameworks, and also by promoting interdisciplinary collaborations, which can drive effective real-world solutions.

## Data Availability

The raw data supporting the conclusions of this article will be made available by the authors, without undue reservation.

## Footnotes

^1^CORA Dataset accessible at: https://networks.skewed.de/net/dblp_author_paper.

^2^DBLP Dataset accessible at: http://konect.cc/networks/dblp-cite/.

^3^Citeseer Dataset accessible at: https://linqs.org/datasets/##citeseer-doc-classification.

^4^Facebook Dataset accessible at: https://networks.skewed.de/?search=Facebook.

^5^Twitter Dataset accessible at: http://konect.cc/networks/munmun_twitterex_ut/.

^6^Flickr Dataset accessible at: https://networks.skewed.de/?search=Flickr.

^7^Zachary's Karate Club Dataset accessible at: https://networks.skewed.de/net/karate.

^8^UCI-HAR Dataset accessible at: https://www.kaggle.com/datasets/drsaeedmohsen/ucihar-dataset.

^9^MNIST Dataset accessible at: https://www.kaggle.com/datasets/hojjatk/mnist-dataset.

^10^20 Newsgroups Dataset accessible at: http://qwone.com/~jason/20Newsgroups/.

^11^1000GP Dataset accessible at: https://www.internationalgenome.org/data.
